# Multivariate Longitudinal Analysis with Bivariate Correlation Test

**DOI:** 10.1371/journal.pone.0159649

**Published:** 2016-08-18

**Authors:** Eric Houngla Adjakossa, Ibrahim Sadissou, Mahouton Norbert Hounkonnou, Gregory Nuel

**Affiliations:** 1 Laboratoire de Probabilités et Modèles Aléatoires /Université Pierre et Marie Curie, Case courrier 188 - 4, Place Jussieu 75252 Paris cedex 05 France; 2 University of Abomey-Calavi, 072 B.P. 50 Cotonou, Republic of Benin; 3 Laboratoire de Biologie et de Physiologie Cellulaires /University of Abomey-Calavi, Cotonou, Republic of Benin; 4 Centre d’Etude et de Recherche sur le Paludisme Associé à la Grossesse et à l’Enfance (CERPAGE), Cotonou, Republic of Benin; University of Amsterdam, NETHERLANDS

## Abstract

In the context of multivariate multilevel data analysis, this paper focuses on the multivariate linear mixed-effects model, including all the correlations between the random effects when the dimensional residual terms are assumed uncorrelated. Using the EM algorithm, we suggest more general expressions of the model’s parameters estimators. These estimators can be used in the framework of the multivariate longitudinal data analysis as well as in the more general context of the analysis of multivariate multilevel data. By using a likelihood ratio test, we test the significance of the correlations between the random effects of two dependent variables of the model, in order to investigate whether or not it is useful to model these dependent variables jointly. Simulation studies are done to assess both the parameter recovery performance of the EM estimators and the power of the test. Using two empirical data sets which are of longitudinal multivariate type and multivariate multilevel type, respectively, the usefulness of the test is illustrated.

## Introduction

In statistical studies, one often needs to analyze data with nested sources of variability: e.g., pupils in classes, employees in companies, repeated measurements in subjects, etc. [1] referred to these type of data as grouped data which are also named multilevel data, hierarchical data or nested data in the literature [2–4]. In the analysis of such data, it is usually illuminating to take account of the variability associated with each level of nesting. There is variability, e.g., between pupils but also between classes. The measurements related to a specific subject (level of nesting) can be correlated, while observations from different subjects are usually independent, and one may draw wrong conclusions if either of these sources of variability is ignored [5]. A series of works in statistical literature focus on the analysis of univariate multilevel data (or univariate grouped data) where a single outcome of interest is analyzed [6–11]. Such analyses are generally simple to deal with due to the availability of many software packages conceived to perform them [12–14]. In practice, many scientific questions of interest require to focus on multiple outcomes, all arising from the same multilevel study, leading to the so-called multivariate multilevel data. For example, to answer some questions of interest, [15] analyzed hearing threshold data (in the Baltimore Longitudinal Study on Aging) [16] which consisted in the longitudinal recording of 22 variables. [17] also studied the joint evolution of HIV RNA and CD4+ T lymphocytes in a cohort of HIV-1 infected patients treated with highly active antiretroviral treatment, by jointly analyzing both markers. [18] used multivariate multilevel regression analysis to investigate individual level determinants of self rated health and happiness, as well as the extent of community level covariation in health and happiness. [19] also used multivariate multilevel analysis to jointly model three commonly used indicators of fear of crime which are: feeling unsafe alone at home after dark, feeling unsafe walking alone after dark and worry about becoming a victim of crime. A variety of works were devoted to joint modeling during the last few decades (see e.g., [20–24]).

These analyses often require a specification of the joint density of all outcomes or, at least, the correlation structure of the data and therefore can lead to the parsimony and/or computation (optimization) problems as well as to numerical difficulties in statistical inference, when the dimension of these outcomes increases. Many analysis strategies were proposed in the statistical literature to circumvent these problems. These strategies generally consist in reducing the dimensionality of the multivariate vector of outcomes and/or in using a small number of latent variables to model correlations within these data. Joint analysis of multivariate multilevel data then requires a trade-off between the increase of the computational complexity and the gain in information.

In this work, we focus on the multivariate linear mixed-effects model, including all the correlations between the random effects along with the independent marginal (dimensional) residuals. The correlations between two dependent variables are then those from the random effects related to these dependent variables. The class of mixed-effects models considered here assumes that both the random effects and the errors (residuals) follow Gaussian distributions. These models are intended for the analysis of multivariate multilevel data in which the dependent variables are continuous.

We use the EM algorithm to estimate the parameters of the model but here, we have two novelties: 1) we suggest a general expression of EM-based estimators which can help in analyzing multivariate longitudinal data as well as the multivariate multilevel data, not of the longitudinal type, and 2) we test the significance of the correlations between the random effects of two dependent variables, using the likelihood ratio test which allows to decide if some dependent variables are significantly correlated or not. By using this bivariate correlation test, the novelty here is the illustration, through empirical data, of some of the consequences of performing separate analyses when a joint analysis is required. Two dependent variables which are found to be uncorrelated after this test will be analyzed with two independent models (or analyzed separately). This strategy may be considered as a way toward the obtaining of a more parsimonious model in high dimension without losing much information. It may also be used in a joint modeling selection procedure.

The paper is organized as follows. In Section 2, contributions of previous works are briefly presented. We also present in this section the EM-based estimators of the parameters of the multivariate linear mixed model. Simulations studies are done in Section 3 where we also discuss the power of the likelihood ratio test which allows to test the significance of the correlation between two response variables. Two illustrations on empirical data are also done in Section 3. The first, concerning bivariate two-level data, is about a study on the effects of school differences on pupils’ progress in Dutch language and arithmetics in the Netherlands. The second illustration concerns a longitudinal study on the immune response to malaria of infants in Benin.

## Materials and Methods

### Previous works

In this Section, we briefly recall the framework of the multivariate multilevel analysis (see for instance, [25, 26]). We can basically distinguish two main approaches to model such data: those which specify the joint distribution of all outcomes without the use of latent structures, and the models using latent structures. We denote by *y*_1_, …, *y*_*m*_ the *m* dependent vectors of interest, and $y={\left(y_{1}^{⊤},\cdots ,y_{m}^{⊤}\right)}^{⊤}$.

#### Modeling methods without latent structures

The first approach which is that of the modeling without latent structures comprises three sub-approaches consisting in a) direct specification of the correlation structure of ***y***, b) analysis without explicit modeling of the correlation structure of ***y*** and c) conditional models.

In the case of direct specification of Cov(***y***), [27] and [28] factorized the covariance matrix of ***y*** by using the Kronecker product in order to have more parsimonious models in the context of fully balanced data. With the same idea of having a parsimonious structure, [29] specified the intra-outcome and inter-outcome correlations, respectively, as follows: $\text{Corr}\left(y_{k}\left(t\right),y_{k}\left(s\right)\right)=\exp\left(\alpha {\left|s-t\right|}^{\theta_{k}}\right)$ and $\text{Corr}\left(y_{k}\left(t\right),y_{k^{\prime }}\left(s\right)\right)=\exp\left({\left(\alpha |s-t|+1\right)}^{\theta_{kk^{\prime }}}\right)$, with *t* and *s* indicating the time, and *k* and *k*′ indicating the dimension. Although these models are useful, they are often too restrictive and may not be realistic in many applications, especially when the data, for example, in the longitudinal studies are unbalanced (i.e. the number of available measurements per subject and the time points at which the measurements were taken often differ from one subject to another). Another class of joint models, specifying directly the joint distribution of ***y***, and whose application is often not straightforward, due to unbalanced data structures is the so-called copula model [30, 31]. Denoting by *F*_*i*_, *i* = 1, …, *m* the cumulative distribution function of the *i*th component of ***y***, *y*_*i*_, a copula model is defined by an *m*-dimensional cumulative distribution function *C*(*u*_1_, …, *u*_*m*_) with uniform marginals such that *F*(*y*_1_, …, *y*_*m*_) = *C*(*u*_1_, …, *u*_*m*_) with (*U*_1_, …, *U*_*m*_) = (*F*_1_(*y*_1_), …, *F*_*m*_(*y*_*m*_)), where *F* is the joint cumulative distribution of ***y***. That is, the joint distribution of ***y*** can be written in terms of its marginal distributions and a copula which describes the dependence structure between its components. While the construction of copulas is mathematically elegant, parameters estimation is often not feasible, especially in high-dimensional situations [26]. One of the rare applications of the copula-based modeling in the multivariate multilevel data analysis framework was proposed by [32], who studied the hemodynamic effect of a new antidepressant on the diastolic blood pressure, the systolic blood pressure and the heart rate of 10 healthy volunteers. They separately modeled, at first, each longitudinal series of response and used a copula to relate the marginal distributions of these responses at each observation time. In a second step, at each observation time, the conditional (on the past) distributions of each response were related using another copula describing the relationship between the corresponding variables. One of the advantages of this approach is that there is no need to use the same family of distributions for all response variables. As [33] used ARIMA process to model the error structure of earnings in a longitudinal data analysis context, time series models can also be used for modeling multivariate multilevel data in order to describe the dynamic dependence between variables and perform forecasting. The most commonly used multivariate time series model, the vector autoregressive (VAR) model which is relatively easy to estimate, is found to be similar to the multivariate multiple linear regression [34] where the errors for different response variables on the same trial are set to be correlated [35]. Other examples of VAR modeling include [36] and [37], but one drawback of the model is that the number of parameters can become very large, potentially leading to estimation problems [38].

Regarding analysis without explicit specification of Cov(***y***), [6] proposed an extension of generalized linear models to the analysis of longitudinal data, where they introduced a class of estimating equations called generalized estimating equations (GEE). GEE estimation ensures consistent estimates of the regression parameters without specifying the joint distribution of a subject’s observations. That is, GEE replaces V[y] by the so-called working covariance matrix *W*(*α*) which depends on an unknown vector *α* to estimate. The related working correlation matrix, *R*(*α*), is also considered. Incorrect choice of *W*(*α*) does not affect the consistency of the regression parameters’ estimators [6]. [39] discussed the use of GEEs with multivariate discrete variables, where focus was on the modeling of the marginal (dimensional) means of these variables and their pairwise associations. The extension of the GEE method to mixed continuous-discrete responses was discussed by [40] and [41]. [42] also avoided the need of explicit modeling of the covariance structure of bivariate longitudinal responses by using SUR [5] and GEE. As pointed out by [43], ambiguities concerning the definition of the working covariance matrix can result in a breakdown of the GEE-based estimation. For example in the longitudinal data analysis, if the true structure of correlation is equicorrelation, ${\left(R_{i}^{*}\right)}_{jk}=\rho$, and that the working structure is autoregressive, (*R*_*i*_)_*jk*_ = *α*^|*j*−*k*|^, there is no solution for $\hat{\alpha }$ when −1/2 ≤ *ρ* < −1/3 [43]. This can be viewed as the major drawback of the GEE method since it can lead to the misspecification of within-subject associations in the context of longitudinal data analysis, for instance. Examples of procedures which bypass the need to explicitly model the underlying covariance structure of ***y*** include [42, 44, 45]. These procedures, generally, consist in regressing each component of ***y*** on relevant covariates of interest, followed by combination of these regression coefficients into a single global estimate of the covariates effect [25].

One way to avoid the direct specification of the joint distribution of ***y*** is to factorize it, leading to the so-called conditional models [46]. For two responses, the joint density *f*(*y*_1_, *y*_2_) can be written as follows:

$$\begin{array}{c} f\left(y_{1},y_{2}\right)=f\left(y_{1}|y_{2}\right)f\left(y_{2}\right)=f\left(y_{2}|y_{1}\right)f\left(y_{1}\right) \end{array}$$ (1)

The choice of the conditioning response is of course arbitrary and requires very careful reflection about plausible associations between components of ***y***. For example, in the specification of a conditional model such as *f*(*y*_1_|*y*_2_), *y*_2_ plays the role of covariate and different choices can lead to completely opposite results and conclusions [47]. In a clinical trial, for example, none of these factorizations will be of interest due to the conditioning on a post-randomization outcome which may partially attenuate the treatment effect on the other [26]. Another drawback of conditional models is that they do not directly lead to marginal inferences. Suppose that scientific interest would be in a comparison of the rate of longitudinal change in average of *y*_1_ and *y*_2_. The factorization *f*(*y*_1_, *y*_2_) = *f*(*y*_1_|*y*_2_)*f*(*y*_2_) directly allows for inferences about the marginal evolution of *y*_2_, but the marginal expectation of *y*_1_ requires computation of $E\left[y_{1}\right]=E[E[y_{1}|y_{2}]]$, which, depending on the actual models, may be far from straightforward [26].

#### Modeling methods using latent structures

The second approach regarding models using latent structures can also be split in two sub-approaches including the strategy based on the reduction of the dimensionality of ***y*** and the mixed-effect models. The general idea of reducing the dimension of ***y*** is to use principal-component type analysis, or a summary function, to first reduce the dimensionality of ***y*** and then, use standard univariate multilevel models for the analysis of the principal factors or the retained summaries of ***y*** [48–52]. Although it is useful, simple to understand and easy to compute, this strategy of dimension reduction has some drawbacks such as the loss of information as discussed by [25] and [26]. [25] used this approach and retained the first principal-component only which explains 31% of the total variation in their data. They found out that the summary function does not have any physical significance and the inference results cannot be interpreted in terms of the effect of the covariates on the original (response) variables. They also found that the method fails to explore the association of the components of ***y*** along time, in the case of longitudinal studies. Furthermore, the method is not applicable in situations where all the components of ***y*** are not measured at the same time point [25], although a possible extension might be the use of functional principal components [53].

Regarding the mixed-effect models, [15], [54], [55], [56] and [57] proposed the use of random-effects models for multivariate longitudinal data. They pointed out that the main disadvantage of joining separate mixed models by allowing their model-specific random effects to be correlated is the increase of the dimension of the total vector of random effects with the number of outcomes, leading to computational problems. To circumvent these problems, [15] noted that all parameters in the joint model can be estimated by fitting all the bivariate models, based on

$$\begin{array}{c} f\left(y_{s},y_{t}\right)=\int \int f\left(y_{s}|\gamma_{s}\right)f\left(y_{t}|\gamma_{t}\right)f\left(\gamma_{s},\gamma_{t}\right)d\gamma_{s}d\gamma_{t} \end{array}$$

for all *m*(*m* − 1)/2 pairs (*y*_*s*_, *y*_*t*_), 1 ≤ *s* < *t* ≤ *m*, resulting from the main multivariate model. Estimators for the main parameters are obtained by averaging over the results from fitting the *m*(*m* − 1)/2 pairwise models. They then showed that the pseudo-likelihood theory can be used to derive the asymptotic distribution of these estimators, and used SAS procedures for mixed models [14] based on the Newton-Raphson algorithm to fit their models, following the approach in [17]. In some multilevel studies, focus is not to directly model ***y***, but a few number of latent variables which cannot be quantified directly (e.g., depression and anxiety), but through measurements of ***y***. In such situations, analysis may be conducted in two steps: the first produces the obtaining of the latent variables and the second proceeds to the joint analysis of these latent variables. For example, [58] proposed a latent factor linear mixed model to capture the joint trend over time of latent variables. the authors reduced, indeed, the high-dimensional responses to low-dimensional latent factors by the factor analysis model, and then used the multivariate linear mixed model to study the longitudinal trends of these latent factors, where the estimates have been done using the EM algorithm. To deal with missing values in multivariate longitudinal analysis using multivariate linear mixed-effects model, [59] proposed multiple imputations using Markov chain Monte Carlo, where they used EM algorithm for the parameters estimation. Here, the authors sped up the EM algorithm by analytically integrating the random effects out of the likelihood function, avoiding to treat them as missing data. [60] used EM based modeling to estimate the parameters of the multivariate linear mixed model under a SAS macro program encoded in IML.

Although the EM algorithm is known to be slow, one of the biggest advantages of this method is that it is not computationally expensive, even with a large number of response variables. In this context, our contribution is the writing of the EM-based estimators in a more general form than those used in [58, 59] and [60]. The expressions of the EM-based estimators used in this paper can easily perform any analysis in the framework of the multivariate multilevel data analysis using multivariate linear mixed-effects model.

Another technique somewhat close to those discussed in [58] is the structural equations-based techniques. For example, [61] developed linear structural equations with latent variables approach. Considering $y={\left(y_{1}^{⊤},y_{2}^{⊤}\right)}^{⊤}$, this approach can be expressed as follows: *y*_*i*_ = *μ*_*i*_ + *G*_*i*_ *η*_*i*_;*i* = 1, 2 and *βη*_1_ = *γη*_2_, where *η*_*i*_, *i* = 1, 2 are the latent variables, *β*(*m* × *m*) and *γ*(*m* × *n*) are coefficient matrices governing the linear relations of all variables involved in the *m* structural equations. *G*_*i*_, *i* = 1, 2 are known matrices. The parameters of the model may be estimated by gradient and quasi-Newton methods, or a Gauss-Newton algorithm that obtains least-squares, generalized least-squares, or maximum likelihood estimates. One modeling strategy which fuses together mixed-effects model and VAR model in order to analyze multivariate multilevel data is the so-called multilevel-VAR method. For example, [62] used the multilevel-VAR model in the context of network inference in psychopathology, where they used the population standard deviation of the person-specific random effects to construct a network representing individual variability. Examples of multilevel-VAR modeling include [63] and [38].

State space models [64] which are useful to investigate the dynamical properties of latent variables can also be used to analyze multivariate multilevel data. For example, [65] introduced an extension of the basic state space model which is flexible and general in the sense of it is applicable to any time series for multiple systems.

Methods for estimating the connectivity maps containing heterogeneity may also be applied to analyze multivariate multilevel data. [66] presented the Group Iterative Multiple Model Estimation (GIMME) approach, which addresses the issue of heterogeneity (the need for individual-level maps) in effective connectivity mapping while capitalizing on shared information to arrive at group inferences. Unlike mixed-effects models, GIMME allows for the structure of the connectivity maps to be unique across individuals [66].

One can also use a nonparametric function *f* to handle the relationship between the components of ***y*** and the covariates [67–69]. This strategy requires also to have sufficient data per subject, in the case of multivariate longitudinal data. Other estimation strategies implemented under softwares and discussed by [70] can perhaps be extended to the multivariate analysis case, when necessary.

Let us finally point out that the software packages which can easily and accurately analyze (jointly) the data of multivariate multilevel type are extremely rare, and one arranges the data and manipulates packages primarily designed for fitting univariate models to handle their analysis. The SabreR [71] package, under the R software [72], which has been devoted to jointly fitting up to three mixed-effects models, with random intercepts only, has been recently removed from the depot. These facts prove by themselves that the analysis of multivariate multilevel data in a single framework is a challenging task. Bayesian-based approaches can be implemented using packages like R2WinBUGS [73] under the R software, and are useful but very time consuming and require a good expertise from the user who can easily be discouraged.

### Model and notations

The model discussed here is the multivariate linear mixed-effects model (or the multivariate linear multilevel model), including all the correlations between the random effects, but the marginal residual terms are assumed to be uncorrelated. For a more general multivariate linear mixed-effects model, the dependent variables are assumed to be correlated, conditional on the random-effects. That is, the marginal residual terms are correlated. In this paper, as in many other works (see for example, [59, 60, 74] and [58]), we assume that conditional on the random-effects, the dependent variables are uncorrelated. In the context of using EM algorithm in estimating the model parameters, this assumption allows to derive the EM-based estimators for the residual variance parameters. If the dimensional residual terms are assumed to be correlated, the EM-based estimators of theirs variance parameters are not easy to deal with and we don’t treat this case here. This model assumes that both the random effects and the residuals follow Gaussian distribution, and is intended for the analysis of multivariate multilevel data in which the dependent variables are continuous. For the sake of simplicity we focus on the bivariate case (*m* = 2) in most of the paper, but the generalization to higher dimensions (*m* > 2) is straightforward. The model is as follows:

$$\begin{array}{ccc} y_{1} & = & X_{1}\beta_{1}+Z_{1}\gamma_{1}+\varepsilon_{1}\\ y_{2} & = & X_{2}\beta_{2}+Z_{2}\gamma_{2}+\varepsilon_{2} \end{array}$$ (2)

$$\begin{array}{c} \gamma =(\begin{array}{c} \gamma_{1}\\ \gamma_{2} \end{array})∼N(0;\Gamma =(\begin{array}{cc} \Gamma_{1} & \Gamma_{12}\\ \Gamma_{12}^{⊤} & \Gamma_{2} \end{array})), \end{array}$$ (3)

$$\begin{array}{c} \varepsilon =(\begin{array}{c} \varepsilon_{1}\\ \varepsilon_{2} \end{array})∼N(0;\Sigma =(\begin{array}{cc} \Sigma_{1} & 0\\ 0 & \Sigma_{2} \end{array}));\;\gamma ⊥\varepsilon \end{array}$$ (4)

For *k* ∈ {1, 2}, *β*_*k*_ and *γ*_*k*_ denote respectively the fixed effects and the random effects vector of covariates, while *ε*_*k*_ is the residual component. *X*_*k*_ is a matrix of covariates and *Z*_*k*_ a covariates-based design matrix. dim(*X*_*k*_) = *N*_*k*_ × *p*_*k*_ and dim(*Z*_*k*_) = *N*_*k*_ × *q*_*k*_, where *N*_*k*_ is the total number of observations in the dimension *k* of the model. *p*_*k*_ and *q*_*k*_ are, respectively, the number of fixed effect related covariates and the number of random effect related covariates in the dimension *k* of the model. If *N*_*k*_ is a constant *N* for any *k*, the index *k* will be removed and *N* will denote the total number of observations in all dimensions of the model. The bold symbols represent parameters of multiple dimensions (i.e. Σ_1_ concerns dimension 1 of the model while **Σ** concerns both dimensions).

Another way to easily understand the model is to express it using the levels of the covariate related to the random-effects. This expression (subject-based version) of the model is, generally, used in the framework of longitudinal data analysis, and lead to EM-based estimators (expressions) which are a particular case of the estimators expressions obtained in Eqs (17), (18) and (19) (for example, see [60]). Denoting by *n* the total number of subjects involved in the longitudinal study, the model can be expressed as follows:

denoting by *i* a subject, for *i* = 1, …, *n*

$$\begin{array}{ccc} y_{1i} & = & X_{1i}\beta_{1}+Z_{1i}\gamma_{1i}+\varepsilon_{1i}\\ y_{2i} & = & X_{2i}\beta_{2}+Z_{2i}\gamma_{2i}+\varepsilon_{2i} \end{array}$$ (5)

with

$$\begin{array}{c} \gamma_{i}=(\begin{array}{c} \gamma_{1i}\\ \gamma_{2i} \end{array})∼N(0;\bar{\Gamma }=(\begin{array}{cc} {\bar{\Gamma }}_{1} & {\bar{\Gamma }}_{12}\\ {\bar{\Gamma }}_{12}^{⊤} & {\bar{\Gamma }}_{2} \end{array})) \end{array}$$ (6)

and

$$\begin{array}{c} \varepsilon_{i}=(\begin{array}{c} \varepsilon_{1i}\\ \varepsilon_{2i} \end{array})∼N(0;\bar{\Sigma }=(\begin{array}{cc} \sigma_{1}^{2}{\text{I}}_{N_{1i}} & 0\\ 0 & \sigma_{2}^{2}{\text{I}}_{N_{2i}} \end{array})) \end{array}$$ (7)

*N*_1*i*_ and *N*_2*i*_ are the dimensions of *y*_1*i*_ and *y*_2*i*_, respectively. Here, we assume that the marginal residuals are homoscedastic ($V\left[\varepsilon_{ki}\right]=\sigma_{k}^{2}I_{N_{ki}},k=1,2$), but the residual covariance matrices can be of full form as in Eq (4). In order to make clear the relation between the model described by Eqs (2), (3) and (4), and its version expressed by Eqs (5), (6) and (7), we propose below a detailed example.

#### Detailed example

We place ourselves in the case of longitudinal data where we observe two response variables *y*_1_ and *y*_2_ which are respectively the weight (kg) and the size (cm) of infants according to the score (V_2_) of the quality of their food as well as the quality score (V_1_) of their mothers’ food. Infants are *n* = 3 girls (sex = F) and boys (sex = M) who are monitored over time. The dataset is presented by Table 1.

**Table 1:** Example of data.

Response variables						
subject	age	sex	V_1_	V_2_	*y*_1_	*y*_2_
1	0	F	63.76	38.16	3.14	47.82
2	4	M	100.88	41.46	4.87	64.02
1	6	F	60.98	41.37	8.43	73.21
3	0	M	93.24	48.76	2.82	44.93
2	7	M	101.95	44.79	8.03	89.54
2	10	M	99.24	48.17	10.08	92.14
1	16	F	NA	44.79	13.96	86.12
3	9	M	88.38	47.91	8.47	86.42

Suppose that the model at each of two dimensions has one random intercept by subject (infant) and one random slope by subject in the direction of the infant’s age (in months). For example, considering an identifiability constraint covering the sex variable whose level F is the reference, the bivariate linear mixed model can be written as follows:

$$\begin{array}{c} y_{1}=\underset{=X_{1}}{\underset{︸}{\left(1\;\text{V}{}_{1}\;\text{sex = M}\right)}}\beta_{1}+Z_{1}\gamma_{1}+\varepsilon_{1}\\ y_{2}=\underset{=X_{2}}{\underset{︸}{\left(1\;\text{V}{}_{2}\;\text{sex = M}\right)}}\beta_{2}+Z_{2}\gamma_{2}+\varepsilon_{2} \end{array}$$ (8)

where, explicitly

$$\begin{array}{c} X_{1}=(\begin{array}{ccc} 1 & 63.76 & 0\\ 1 & 100.88 & 1\\ 1 & 60.98 & 0\\ 1 & 93.24 & 1\\ 1 & 101.95 & 1\\ 1 & 99.24 & 1\\ 1 & 88.38 & 1 \end{array});X_{2}=(\begin{array}{ccc} 1 & 38.16 & 0\\ 1 & 41.46 & 1\\ 1 & 41.37 & 0\\ 1 & 48.76 & 1\\ 1 & 44.79 & 1\\ 1 & 48.17 & 1\\ 1 & 44.79 & 0\\ 1 & 47.91 & 1 \end{array}) \end{array}$$ (9)

$$\begin{array}{c} Z_{1}=(\begin{array}{cccccc} 1 & 0 & 0 & 0 & 0 & 0\\ 0 & 0 & 1 & 4 & 0 & 0\\ 1 & 6 & 0 & 0 & 0 & 0\\ 0 & 0 & 0 & 0 & 1 & 0\\ 0 & 0 & 1 & 7 & 0 & 0\\ 0 & 0 & 1 & 10 & 0 & 0\\ 0 & 0 & 0 & 0 & 1 & 9 \end{array});Z_{2}=(\begin{array}{cccccc} 1 & 0 & 0 & 0 & 0 & 0\\ 0 & 0 & 1 & 4 & 0 & 0\\ 1 & 6 & 0 & 0 & 0 & 0\\ 0 & 0 & 0 & 0 & 1 & 0\\ 0 & 0 & 1 & 7 & 0 & 0\\ 0 & 0 & 1 & 10 & 0 & 0\\ 1 & 16 & 0 & 0 & 0 & 0\\ 0 & 0 & 0 & 0 & 1 & 9 \end{array}) \end{array}$$ (10)

$$\begin{array}{c} \dim\left(X_{1}\right)=7\times 3\;\text{and}\;\dim\left(X_{2}\right)=8\times 3;\;\dim\left(Z_{1}\right)=7\times 6\;\text{and}\;\dim\left(Z_{2}\right)=8\times 6 \end{array}$$

In the present example we have dim(*X*_1_) ≠ dim(*X*_2_) and dim(*Z*_1_) ≠ dim(*Z*_2_) due to the presence of the NA (Not Available) within the values of the variable V_1_. Removing information related to this NA in the dimension 1 of the model does not affect its dimension 2.

$$\begin{array}{c} \Gamma =(\begin{array}{cc} \Gamma_{1} & \Gamma_{12}\\ \Gamma_{12}^{⊤} & \Gamma_{2} \end{array}) \end{array}$$

with,

$$\begin{array}{c} \Gamma_{1}=(\begin{array}{cccccc} \eta_{1}^{2} & \rho_{\eta }\eta_{1}\eta_{2} & . & . & . & .\\ \rho_{\eta }\eta_{1}\eta_{2} & \eta_{2}^{2} & . & . & . & .\\ . & . & \eta_{1}^{2} & \rho_{\eta }\eta_{1}\eta_{2} & . & .\\ . & . & \rho_{\eta }\eta_{1}\eta_{2} & \eta_{2}^{2} & . & .\\ . & . & . & . & \eta_{1}^{2} & \rho_{\eta }\eta_{1}\eta_{2}\\ . & . & . & . & \rho_{\eta }\eta_{1}\eta_{2} & \eta_{2}^{2} \end{array}) \end{array}$$ (11)

$$\begin{array}{c} \Gamma_{2}=(\begin{array}{cccccc} \tau_{1}^{2} & \rho_{\tau }\tau_{1}\tau_{2} & . & . & . & .\\ \rho_{\tau }\tau_{1}\tau_{2} & \tau_{2}^{2} & . & . & . & .\\ . & . & \tau_{1}^{2} & \rho_{\tau }\tau_{1}\tau_{2} & . & .\\ . & . & \rho_{\tau }\tau_{1}\tau_{2} & \tau_{2}^{2} & . & .\\ . & . & . & . & \tau_{1}^{2} & \rho_{\tau }\tau_{1}\tau_{2}\\ . & . & . & . & \rho_{\tau }\tau_{1}\tau_{2} & \tau_{2}^{2} \end{array}) \end{array}$$ (12)

$$\begin{array}{c} \Gamma_{12}=(\begin{array}{cccccc} \rho_{\eta_{1}\tau_{1}}\eta_{1}\tau_{1} & \rho_{\eta_{1}\tau_{2}}\eta_{1}\tau_{2} & . & . & . & .\\ \rho_{\eta_{2}\tau_{1}}\eta_{2}\tau_{1} & \rho_{\eta_{2}\tau_{2}}\eta_{2}\tau_{2} & . & . & . & .\\ . & . & \rho_{\eta_{1}\tau_{1}}\eta_{1}\tau_{1} & \rho_{\eta_{1}\tau_{2}}\eta_{1}\tau_{2} & . & .\\ . & . & \rho_{\eta_{2}\tau_{1}}\eta_{2}\tau_{1} & \rho_{\eta_{2}\tau_{2}}\eta_{2}\tau_{2} & . & .\\ . & . & . & . & \rho_{\eta_{1}\tau_{1}}\eta_{1}\tau_{1} & \rho_{\eta_{1}\tau_{2}}\eta_{1}\tau_{2}\\ . & . & . & . & \rho_{\eta_{2}\tau_{1}}\eta_{2}\tau_{1} & \rho_{\eta_{2}\tau_{2}}\eta_{2}\tau_{2} \end{array}) \end{array}$$ (13)

*ρ*_*η*_, *ρ*_*τ*_, *ρ*_*η*_1_*τ*_1__, *ρ*_*η*_1_*τ*_2__, *ρ*_*η*_2_*τ*_1__, and *ρ*_*η*_2_*τ*_2__ lie in [−1, 1]. All other parameters involved in Γ_1_, Γ_2_ and Γ_12_ are positive real numbers.

Referring to the subject-based version of the model,

$$\begin{array}{c} {\bar{\Gamma }}_{1}=(\begin{array}{cc} \eta_{1}^{2} & \rho_{\eta }\eta_{1}\eta_{2}\\ \rho_{\eta }\eta_{1}\eta_{2} & \eta_{2}^{2} \end{array}),\;{\bar{\Gamma }}_{2}=(\begin{array}{cc} \tau_{1}^{2} & \rho_{\tau }\tau_{1}\tau_{2}\\ \rho_{\tau }\tau_{1}\tau_{2} & \tau_{2}^{2} \end{array}),\;{\bar{\Gamma }}_{12}=(\begin{array}{cc} \rho_{\eta_{1}\tau_{1}}\eta_{1}\tau_{1} & \rho_{\eta_{1}\tau_{2}}\eta_{1}\tau_{2}\\ \rho_{\eta_{2}\tau_{1}}\eta_{2}\tau_{1} & \rho_{\eta_{2}\tau_{2}}\eta_{2}\tau_{2} \end{array}), \end{array}$$

$$\begin{array}{c} V\left(\gamma_{1}\right)={\bar{\Gamma }}_{1},\;V\left(\gamma_{2}\right)={\bar{\Gamma }}_{2},\;\text{and}\;\text{Cov}\left(\gamma_{1},\gamma_{2}\right)={\bar{\Gamma }}_{12}. \end{array}$$ (14)

Then,

$$\begin{array}{c} \Gamma_{1}=\text{diag}\left({\bar{\Gamma }}_{1},\cdots ,{\bar{\Gamma }}_{1}\right),\;\Gamma_{2}=\text{diag}\left({\bar{\Gamma }}_{2},\cdots ,{\bar{\Gamma }}_{2}\right),\;\text{and}\;\Gamma_{12}=\text{diag}\left({\bar{\Gamma }}_{12},\cdots ,{\bar{\Gamma }}_{12}\right). \end{array}$$ (15)

### EM estimation

Let *θ* be the vector of unknown parameters in *β*_1_, *β*_2_, **Γ**, Σ_1_, Σ_2_. The EM algorithm requires an initial value of *θ* and some expressions (estimators) to update until convergence. In the next two subsections we provide these estimators, their initial values and the stopping criterion.

#### EM-based estimators of parameters

**Theorem 1**. *Suppose that* $y={\left(y_{1}^{⊤},y_{2}^{⊤}\right)}^{⊤}$ *satisfies the model based on Eqs* (2), (3) *and* (4) *and θ the vector of its unknown parameters while* *θ*_old_ *is the previous value of θ provided by the EM algorithm. Let* *f*(***y***,**γ**|*θ*) *be the joint density function of ***y*** and **γ** given* *θ*, *and* $Q\left(\theta |\theta_{\mathrm{old}}\right)=E[\log f\left(y,\gamma |\theta \right)|y,\theta_{\mathrm{old}}]$. *Let M be the mapping* $\theta_{\mathrm{old}}\mapsto M\left(\theta_{\mathrm{old}}\right)=\hat{\theta }$ *such that*:

$$\begin{array}{c} M\left(\theta_{\mathrm{old}}\right)=\underset{\theta }{\text{arg max}}\;Q\left(\theta |\theta_{\mathrm{old}}\right) \end{array}$$ (16)

*Then, the EM-based estimator of θ, i.e.* $\hat{\theta }$, *is expressed through*:

*for* *k* ∈ {1, …, *m*},

$$\begin{array}{c} {\hat{\beta }}_{k}={\left(X_{k}^{⊤}\Sigma_{k}^{-1}X_{k}\right)}^{-1}X_{k}^{⊤}\Sigma_{k}^{-1}(y_{k}-Z_{k}E\left[\gamma_{k}|y,\theta_{\mathrm{old}}\right]), \end{array}$$ (17)

$$\begin{array}{c} \hat{\Gamma }=V\left[\gamma |y,\theta_{\mathrm{old}}\right]+E\left[\gamma |y,\theta_{\mathrm{old}}\right]E{\left[\gamma |y,\theta_{\mathrm{old}}\right]}^{⊤}, \end{array}$$ (18)

$$\begin{array}{c} {\hat{\Sigma }}_{k}=Z_{k}V\left[\gamma_{k}|y,\theta_{\mathrm{old}}\right]Z_{k}^{⊤}+\left(y_{k}-X_{k}\beta_{k}-Z_{k}E\left[\gamma_{k}|y,\theta_{\mathrm{old}}\right]\right){\left(y_{k}-X_{k}\beta_{k}-Z_{k}E\left[\gamma_{k}|y,\theta_{\mathrm{old}}\right]\right)}^{⊤}, \end{array}$$ (19)

*where*,

$$\begin{array}{c} E\left[\gamma_{k}|y,\theta_{\mathrm{old}}\right]=\mathrm{Cov}\left(\gamma_{k},y|\theta_{\mathrm{old}}\right)V{\left[y|\theta_{\mathrm{old}}\right]}^{-1}\left(y-E\left[y|\theta_{\mathrm{old}}\right]\right), \end{array}$$ (20)

$$\begin{array}{c} V\left[\gamma_{k}|y,\theta_{\mathrm{old}}\right]=\Gamma_{k}-\mathrm{Cov}\left(\gamma_{k},y|\theta_{\mathrm{old}}\right)V{\left[y|\theta_{\mathrm{old}}\right]}^{-1}\mathrm{Cov}{\left(\gamma_{k},y|\theta_{\mathrm{old}}\right)}^{⊤} \end{array}$$ (21)

*and*

$$\begin{array}{c} V\left[y|\theta_{\mathrm{old}}\right]=(\begin{array}{cc} Z_{1}\Gamma_{1}Z_{1}^{⊤}+\Sigma_{1} & Z_{1}\Gamma_{12}Z_{2}^{⊤}\\ Z_{2}\Gamma_{12}^{⊤}Z_{1}^{⊤} & Z_{2}\Gamma_{2}Z_{2}^{⊤}+\Sigma_{2} \end{array}),\;\mathrm{Cov}\left(\gamma ,y|\theta_{\mathrm{old}}\right)={\left(\begin{array}{cc} Z_{1}\Gamma_{1} & Z_{1}\Gamma_{12}\\ Z_{2}\Gamma_{12}^{⊤} & Z_{2}\Gamma_{2} \end{array}\right)}^{⊤}, \end{array}$$ (22)

$$\begin{array}{c} \mathrm{Cov}\left(\gamma_{1},y|\theta_{\mathrm{old}}\right)={\left(\begin{array}{c} Z_{1}\Gamma_{1}\\ Z_{2}\Gamma_{12}^{⊤} \end{array}\right)}^{⊤},\;\mathrm{Cov}\left(\gamma_{2},y|\theta_{\mathrm{old}}\right)={\left(\begin{array}{c} Z_{1}\Gamma_{12}\\ Z_{2}\Gamma_{2} \end{array}\right)}^{⊤}. \end{array}$$ (23)

*proof*. For *k* ∈ {1, …, *m*}, ${\hat{\beta }}_{k}$, ${\hat{\Sigma }}_{k}$ and $\hat{\Gamma }$ optimize the quantity:

$$\begin{array}{c} Q\left(\theta |\theta_{\text{old}}\right)=E[\log f\left(y,\gamma |\theta \right)|y,\theta_{\text{old}}] \end{array}$$ (24)

where *f*(***y***,**γ**|*θ*) is the joint density function of the observed data ***y*** and the random effect **γ**. In the case of *m* = 2, we have:

$$\begin{array}{ccc} f(y,\gamma |\theta ) & = & f(y|\gamma ,\theta )f(\gamma |\theta )\\ & = & f\left(y_{1}|\gamma_{1},\theta \right)f\left(y_{2}|\gamma_{2},\theta \right)f\left(\gamma |\theta \right)\\ & = & {\left(2\pi \right)}^{-(N_{1}+N_{2}+q)/2}{|\Sigma_{1}|}^{-1/2}{|\Sigma_{2}|}^{-1/2}{\left|\Gamma \right|}^{-1/2}\exp\{-\frac{1}{2}\gamma^{⊤}\Gamma^{-1}\gamma \\ & & -\frac{1}{2}{\left(y_{1}-X_{1}\beta_{1}-Z_{1}\gamma_{1}\right)}^{⊤}\Sigma_{1}^{-1}\left(y_{1}-X_{1}\beta_{1}-Z_{1}\gamma_{1}\right)\\ & & -\frac{1}{2}{\left(y_{2}-X_{2}\beta_{2}-Z_{2}\gamma_{2}\right)}^{⊤}\Sigma_{2}^{-1}\left(y_{2}-X_{2}\beta_{2}-Z_{2}\gamma_{2}\right)\} \end{array}$$ (25)

Since *f* is a multivariate Gaussian, using the dominated convergence theorem and the derivative under the integral sign, the differential of *Q*(*θ*|*θ*_old_) yields:

$$\begin{array}{ccc} \text{d}Q(\theta |\theta_{\text{old}}) & = & E[-\frac{1}{2}\text{tr}\{\Sigma_{1}^{-1}\text{d}\Sigma_{2}+\Sigma_{2}^{-1}\text{d}\Sigma_{2}+\Gamma^{-1}\text{d}\Gamma \}\\ & & -\frac{1}{2}\text{tr}\{-2{\left(y_{1}-X_{1}\beta_{1}-Z_{1}\gamma_{1}\right)}^{⊤}\Sigma_{1}^{-1}X_{1}\text{d}\beta_{1}\\ & & -\Sigma_{1}^{-1}\left(y_{1}-X_{1}\beta_{1}-Z_{1}\gamma_{1}\right){\left(y_{1}-X_{1}\beta_{1}-Z_{1}\gamma_{1}\right)}^{⊤}\Sigma_{1}^{-1}\text{d}\Sigma_{1}\}\\ & & -\frac{1}{2}\text{tr}\{-2{\left(y_{2}-X_{2}\beta_{2}-Z_{2}\gamma_{2}\right)}^{⊤}\Sigma_{2}^{-1}X_{2}\text{d}\beta_{2}\\ & & -\Sigma_{2}^{-1}\left(y_{2}-X_{2}\beta_{2}-Z_{2}\gamma_{2}\right){\left(y_{2}-X_{2}\beta_{2}-Z_{2}\gamma_{2}\right)}^{⊤}\Sigma_{2}^{-1}\text{d}\Sigma_{2}\}\\ & & -\frac{1}{2}\text{tr}\{-\gamma^{⊤}\Gamma^{-1}\text{d}\Gamma \Gamma^{-1}\gamma \}|y,\theta_{\text{old}}] \end{array}$$ (26)

$$\begin{array}{ccc} & = & -\frac{1}{2}\text{tr}\{\Sigma_{1}^{-1}\text{d}\Sigma_{2}+\Sigma_{2}^{-1}\text{d}\Sigma_{2}+\Gamma^{-1}\text{d}\Gamma \}\\ & & +\text{tr}{\left(y_{1}-X_{1}\beta_{1}-Z_{1}E[\gamma_{1}|y,\theta_{\text{old}}]\right)}^{⊤}\Sigma_{1}^{-1}X_{1}\text{d}\beta_{1}\\ & & +\frac{1}{2}\text{tr}\{\Sigma_{1}^{-1}E\left[{\left(y_{1}-X_{1}\beta_{1}-Z_{1}\gamma_{1}\right)}^{⊤}\left(y_{1}-X_{1}\beta_{1}-Z_{1}\gamma_{1}\right)|y,\theta_{\text{old}}\right]\Sigma_{1}^{-1}\text{d}\Sigma_{1}\}\\ & & +\text{tr}{\left(y_{2}-X_{2}\beta_{2}-Z_{2}E[\gamma_{2}|y,\theta_{\text{old}}]\right)}^{⊤}\Sigma_{2}^{-1}X_{2}\text{d}\beta_{2}\\ & & +\frac{1}{2}\text{tr}\{\Sigma_{2}^{-1}E\left[{\left(y_{2}-X_{2}\beta_{2}-Z_{2}\gamma_{2}\right)}^{⊤}\left(y_{2}-X_{2}\beta_{2}-Z_{2}\gamma_{2}\right)|y,\theta_{\text{old}}\right]\Sigma_{2}^{-1}\text{d}\Sigma_{2}\}\\ & & +\text{tr}\frac{1}{2}\{\Gamma^{-1}E\left[\gamma \gamma^{⊤}|y,\theta_{\text{old}}\right]\Gamma^{-1}\text{d}\Gamma \} \end{array}$$ (27)

Partial derivatives of *Q*(*θ*|*θ*_old_) yield:

for *k* ∈ {1, …, *m*},

$$\begin{array}{c} \frac{\partial Q(\theta |\theta_{\text{old}})}{\partial \beta_{k}}={\left(y_{k}-X_{k}\beta_{k}-Z_{k}E[\gamma_{k}|y,\theta_{\text{old}}]\right)}^{⊤}\Sigma_{k}^{-1}X_{k}, \end{array}$$

$$\begin{array}{c} \frac{\partial Q(\theta |\theta_{\text{old}})}{\partial \Sigma_{k}}=-\frac{1}{2}\Sigma_{k}^{-1}+\Sigma_{k}^{-1}E\left[{\left(y_{k}-X_{k}\beta_{k}-Z_{k}\gamma_{k}\right)}^{⊤}\left(y_{k}-X_{k}\beta_{k}-Z_{k}\gamma_{k}\right)|y,\theta_{\text{old}}\right]\Sigma_{k}^{-1} \end{array}$$

and

$$\begin{array}{c} \frac{\partial Q(\theta |\theta_{\text{old}})}{\partial \Gamma }=\frac{1}{2}(\Gamma^{-1}E\left[\gamma \gamma^{⊤}|y,\theta_{\text{old}}\right]\Gamma^{-1}-\Gamma^{-1}). \end{array}$$

We then get EM-based estimators by setting these partial derivatives equal to zero. $E[\gamma_{k}|y,\theta_{\text{old}}]$ and $V[\gamma_{k}|y,\theta_{\text{old}}]$ are straightforward to get since ${\left(\gamma_{k}^{⊤},y^{⊤}\right)}^{⊤}$ is a multivariate Gaussian.

#### Initialization and stopping criterion of the algorithm

Various ways exist for obtaining starting values for ${\hat{\beta }}_{k}$, $\hat{\Gamma }$, ${\hat{\Sigma }}_{k},\;\text{for}\;k=1,\cdots ,m$. Taking inspiration from [75] and [60], we have separately fitted each dimension of the model by using the lme4 package [76] under the R software and have used marginal estimated parameters to initialize ${\hat{\beta }}_{k}$ and ${\hat{\Sigma }}_{k}$. We then keep the expected random effects $\tilde{\gamma }=\hat{E}\left[\gamma |y\right]$ to initialize $\hat{\Gamma }$ by

$$\begin{array}{c} \frac{1}{n-1}\sum_{i=1}^{n}{\tilde{\gamma }}_{i}{\tilde{\gamma }}_{i}^{⊤} \end{array}$$ (28)

The stopping criterion is related to the relative error of the components of *θ* as follows:

$$\begin{array}{c} \max_{j}|\frac{\theta_{j}^{\left(r\right)}-\theta_{j}^{\left(r+1\right)}}{\theta_{j}^{\left(r+1\right)}}|<\text{tol} \end{array}$$ (29)

where (*r*) is the iteration index and *θ*_*j*_ the *j*th component of *θ*. tol = 10^−5^ seems to work well in practice.

### Test of the significance of $\hat{\text{Cor}}\left(\gamma_{1},\gamma_{2}\right)$

After the calculation of **Γ** on dataset, we sometimes need to investigate if the correlation between marginal random effects is statistically significant, by testing *H*_0_: Cor(*γ*_1_, *γ*_2_) = **0** against *H*_1_: Cor(*γ*_1_, *γ*_2_) ≠ **0**. The result of this test can help to decide if the bivariate analysis is justified or not. We perform the likelihood ratio (LR) test to choose between *H*_0_ and *H*_1_. We calculated *S*, the statistic of the likelihood ratio test.

$$\begin{array}{c} S=-2\log(\frac{L(\theta |\text{data,}\;H_{0})}{L(\theta |\text{data,}\;H_{1})}) \end{array}$$ (30)

where $L(\theta |\text{data,}\;H_{0})$ and $L(\theta |\text{data,}\;H_{1})$ are the likelihood of *θ* under *H*_0_ and *H*_1_, respectively. Under suitable and standard conditions, *S* ∼ *χ*^2^(*df*), asymptotically, under *H*_0_ [77]. With *df* the difference in the number of parameters between $L(\theta |\text{data,}\;H_{0})$ and $L(\theta |\text{data,}\;H_{1})$.

## Results and Discussion

### Simulation studies

In this section, simulation studies are used to investigate the computational properties of the EM-based estimators. For the sake of simplicity, these simulation studies are conducted using simulated bivariate longitudinal data sets. Through these studies, we pursue two objectives: the first is to assess the accuracy of parameter estimates and the second is to analyze the power of the likelihood ratio test performed via these EM-based estimators. In the following paragraph, we explain how we choose the parameters that have been used to simulate the working longitudinal data sets.

#### The working data sets

We suppose that we are following up a sample of subjects where the goal is to evaluate how the growth of the weight and the height of the individuals of this population are jointly explained by the sex, the score of nutrition (Nscore) and the age. We randomly choose through a uniform distribution the score of nutrition between 20 and 50, and the age between 18 and 37, using the R software. All the analysis in this paper are done using the R software. The subject’s sex is also randomly chosen. The model under which we simulate the data sets is the following:

*n* indicating the total number of subjects, for *i* = 1, …, *n*

$$\begin{array}{ccc} {\text{weight}}_{i} & = & \left(1_{n_{i}},{\text{sex}}_{i},{\text{Nscore}}_{i},{\text{age}}_{i}\right)\beta_{1}+\left(1_{n_{i}},{\text{Nscore}}_{i}\right)\gamma_{1i}+\varepsilon_{1i}\\ {\text{height}}_{i} & = & \left(1_{n_{i}},{\text{sex}}_{i},{\text{Nscore}}_{i},{\text{age}}_{i}\right)\beta_{2}+\left(1_{n_{i}},{\text{Nscore}}_{i}\right)\gamma_{2i}+\varepsilon_{2i} \end{array}$$ (31)

with

$$\begin{array}{c} \gamma_{i}=(\begin{array}{c} \gamma_{1i}\\ \gamma_{2i} \end{array})∼N(0,\bar{\Gamma }),\varepsilon_{1i}∼N(0,\sigma_{1}^{2}{\text{I}}_{n_{i}}),\varepsilon_{2i}∼N(0,\sigma_{2}^{2}{\text{I}}_{n_{i}}),\gamma_{i}⊥\varepsilon_{1i}⊥\varepsilon_{2i} \end{array}$$ (32)

The random effect related to the dependent variable ‘weight’ or ‘height’ is a vector composed by one random intercept and one random slope in the direction of the covariate ‘Nscore’. The total number of observations is denoted by *N*.

We randomly choose *β*_1_, *β*_2_, *σ*_1_ and *σ*_2_ whose values are in the first column of Table 2. $\bar{\Gamma }$ is also randomly chosen such that it is positive definite, with the following form:

$$\begin{array}{c} \bar{\Gamma }=(\begin{array}{cccc} \eta_{1}^{2} & \rho_{\eta }\eta_{1}\eta_{2} & \rho \eta_{1}\tau_{1} & \rho \eta_{1}\tau_{2}\\ \rho_{\eta }\eta_{1}\eta_{2} & \eta_{2}^{2} & \rho \eta_{2}\tau_{1} & \rho \eta_{2}\tau_{2}\\ \rho \eta_{1}\tau_{1} & \rho \eta_{2}\tau_{1} & \tau_{1}^{2} & \rho_{\tau }\tau_{1}\tau_{2}\\ \rho \eta_{1}\tau_{2} & \rho \eta_{2}\tau_{2} & \rho_{\tau }\tau_{1}\tau_{2} & \tau_{2}^{2} \end{array}) \end{array}$$ (33)

The covariance between the random effects *γ*_1_ and *γ*_2_ is set, intentionally,

$$\begin{array}{c} Cov\left(\gamma_{1},\gamma_{2}\right)=\rho (\begin{array}{cc} \eta_{1}\tau_{1} & \eta_{1}\tau_{2}\\ \eta_{2}\tau_{1} & \eta_{2}\tau_{2} \end{array}) \end{array}$$ (34)

in order to be able to decrease or increase the correlation between the marginal random effects *γ*_1_ and *γ*_2_, by changing the value of *ρ*, without losing the positive definiteness of $\bar{\Gamma }$. This property of $\bar{\Gamma }$ will be used to assess the power of the likelihood ratio test through simulations, by changing the value of *ρ*. We simulate 1000 data sets with *ρ* = 0.8, in order to assess the accuracy of estimates using the EM-based estimators. With *ρ* = 0.8, the randomly chosen $\bar{\Gamma }$ is

$$\begin{array}{c} \bar{\Gamma }=(\begin{array}{cccc} 27.77 & 18.80 & 41.70 & 4.93\\ 18.80 & 36.00 & 47.47 & 5.62\\ 41.70 & 47.47 & 97.81 & 8.91\\ 4.93 & 5.62 & 8.91 & 1.37 \end{array}) \end{array}$$ (35)

**Table 2:** Comparative table of true values of parameters and estimates based on 1000 replications using true values of parameters.

Parameter	Value	Empirical mean	Empirical Sd	Bias
*β*_1_	50.67	50.669	0.763	0.000
	-4.80	-4.779	0.811	0.021
	14.00	14.012	0.345	0.012
	2.70	2.700	0.016	0.000
*β*_2_	13.20	13.263	1.077	0.063
	-2.80	-2.796	1.186	0.003
	27.00	27.000	0.068	0.000
	1.70	1.699	0.019	0.000
*σ*_1_	5.80	5.796	0.062	0.003
*σ*_2_	7.60	7.602	0.082	0.002

#### Empirical accuracy of the estimates

The 1000 data sets simulated in order to assess the accuracy of the estimates performed using the EM-based estimators contain *N* = 5000 observations provided by *n* = 300 independent subjects.

The mean and the standard deviation of the 1000 estimates are presented, respectively, in the second and the third column of the Table 2. The bias of the parameter estimates, which is the absolute difference between the true value of the parameter and the mean of the 1000 estimates, is calculated as measure of performance. These bias are contained in the forth column of the Table 2.

$\hat{E}\left[\hat{\bar{\Gamma }}\right]$, ${\hat{\sigma }}_{\hat{\bar{\Gamma }}}$ and $\text{Bias}(\hat{\bar{\Gamma }})$ (Eqs (36), (37)) contain, respectively, the empirical mean, the empirical standard deviation and the empirical bias of $\bar{\Gamma }$.

$$\begin{array}{c} \hat{E}\left[\hat{\bar{\Gamma }}\right]=(\begin{array}{cccc} 27.86 & 18.71 & 41.19 & 4.93\\ 18.71 & 35.73 & 47.09 & 5.58\\ 41.19 & 47.09 & 95.93 & 8.87\\ 4.93 & 5.58 & 8.87 & 1.36 \end{array});\;{\hat{\sigma }}_{\hat{\bar{\Gamma }}}=(\begin{array}{cccc} 5.32 & 2.99 & 6.37 & 0.63\\ 2.98 & 2.86 & 5.11 & 0.50\\ 6.37 & 5.11 & 13.73 & 0.97\\ 0.63 & 0.50 & 0.97 & 0.11 \end{array}) \end{array}$$ (36)

$$\begin{array}{c} \text{Bias}\left(\hat{\bar{\Gamma }}\right)(\begin{array}{cccc} 0.085 & 0.090 & 0.515 & 0.001\\ 0.089 & 0.265 & 0.382 & 0.045\\ 0.514 & 0.382 & 1.881 & 0.039\\ 0.001 & 0.045 & 0.039 & 0.007 \end{array}) \end{array}$$ (37)

The bias contained in the estimates of *β*_*k*_ and *σ*_*k*_ ranges from 0.000 to 0.063 (Table 2), and the bias contained in the estimates of the entries of $\bar{\Gamma }$ ranges from 0.001 to 1.881 (Eq 37). These results show that ${\hat{\beta }}_{k}$ and ${\hat{\sigma }}_{k}$ (i.e. ${\hat{\Sigma }}_{k}$) seem unbiased when $\hat{\bar{\Gamma }}$ is biased.

**Fig 1:**
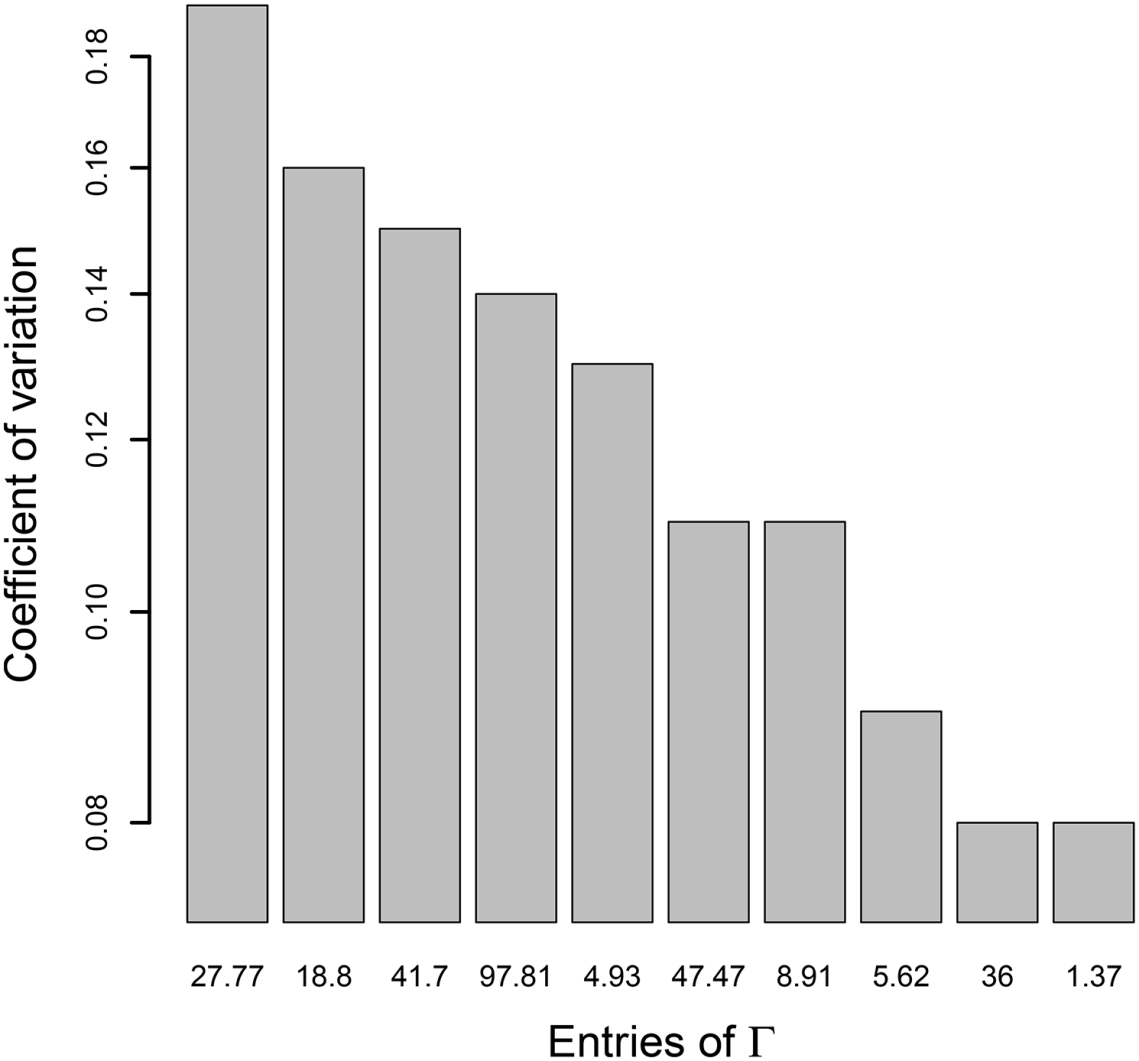
Coefficients of variation of entries of Γ. *N* = 5000 observations and *n* = 300 subjects.

The estimates of $\bar{\Gamma }$ appear to be poorer than the estimates of all other parameters. In order to investigate which entries of $\bar{\Gamma }$ are particularly poorly estimated, we calculate the coefficients of variation (CV) of these entries. The CV computed here is obtained by dividing the standard deviation of the estimates by the true value of each entry of $\bar{\Gamma }$. The CVs give an idea of the variability of estimates around the true values and enable to compare these variabilities between them. A particularly large value of CV could lead us to suspect that the corresponding input is particularly poorly estimated. Here, the CV ranges from 0.08 to 0.19, and is represented by the Fig 1 for more visibility. Given these CV values, it seems that none of the entries of $\bar{\Gamma }$ is particularly poorly estimated.

#### Deep investigation on the estimates’ accuracy

Here, we compute the Mean Square Error (MSE) of the EM-based estimators with *N* = 600,1000 and *N* = 3000 across *n* = 50, 60, 100 and 300 to investigate how both values of *n* and *N* affect the quality of the estimates. For each value of n and N, we simulate 1000 data sets on which we estimate the model parameters and compute the MSE of these estimates.

Without surprise, Table 3 shows that the quality of estimates is clearly improved when both *n* and *N* grow. Estimations performed on dataset containing *N* = 3000 observations are more accurate than those performed with *N* = 600, observing the maximum value of the MSE in each case. For *N* = 600, information contained in Table 3 shows that the MSE related to *n* = 60 (60 subjects) are better than those related to *n* = 300. This result suggests a good tradeoff between the number of subjects and the total number of observations in order to have accurate estimates, especially if the number of observations is not very high. Once again, it appears that $\hat{\Gamma }$ (Table 3) has the highest MSE for all values of *n* and *N*.

**Table 3:** Mean Square Error of EM-based estimator with 95% CI estimated on 1000 replications for various values of *n* and *N*.

Parameter	*n*	*N* = 600	*N* = 1000	*N* = 3000
*β*_1_	50	1.27 (0.01 - 4.64)	1.10 (0.00 - 3.83)	0.71 (0.00 - 2.57)
	60	1.38 (0.00 - 5.56)	0.99 (0.00 - 3.90)	0.65 (0.00 - 2.44)
	100	1.31 (0.00 - 4.79)	0.87 (0.00 - 3.41)	0.46 (0.00 - 1.58)
	300	1.64 (0.00 - 5.93)	0.80 (0.00 - 3.23)	0.29 (0.00 - 1.13)
*β*_2_	50	2.13 (0.01 - 7.22)	1.73 (0.00 - 6.21)	1.08 (0.00 - 4.17)
	60	2.12 (0.00 - 8.06)	1.63 (0.01 - 6.66)	0.95 (0.00 - 3.52)
	100	1.88 (0.00 - 7.14)	1.29 (0.00 - 4.76)	0.62 (0.00 - 2.24)
	300	2.30 (0.00 - 8.54)	1.23 (0.00 - 4.57)	0.43 (0.00 - 1.65)
*σ*_1_	50	0.03 (0.00 - 0.10)	0.10 (0.00 - 0.07)	0.01 (0.00 - 0.02)
	60	0.04 (0.00 - 0.14)	0.02 (0.00 - 0.07)	0.01 (0.00 - 0.02)
	100	0.04 (0.00 - 0.14)	0.02 (0.00 - 0.08)	0.01 (0.00 - 0.02)
	300	0.06 (0.00 - 0.22)	0.03 (0.00 - 0.11)	0.01 (0.00 - 0.02)
*σ*_2_	50	0.05 (0.00 - 0.18)	0.03 (0.00 - 0.12)	0.01 (0.00 - 0.03)
	60	0.06 (0.00 - 0.21)	0.03 (0.00 - 0.12)	0.01 (0.00 - 0.03)
	100	0.06 (0.00 - 0.24)	0.04 (0.00 - 0.15)	0.01 (0.00 - 0.03)
	300	0.09 (0.00 - 0.36)	0.04 (0.00 - 0.18)	0.01 (0.00 - 0.05)
$\bar{\Gamma }$	50	400.91 (1.52 - 1274.32)	670.46 (2.36 - 2536.73)	489.07 (2.02 - 1840.60)
	60	706.45 (2.58 - 2497.42)	620.34 (2.70 - 2293.58)	408.79 (0.85 - 1597.77)
	100	701.50 (3.79 - 2747.70)	477.55 (2.08 - 1839.26)	283.25 (1.97 - 1023.77)
	300	798.28 (3.65 - 2603.65)	547.83 (3.03 - 1721.46)	199.54 (0.81 - 736.08)

#### The bivariate likelihood ratio test

Considering the random effects covariance matrix $\bar{\Gamma }$ (see Eq (35)), the related correlation matrix is

$$\begin{array}{c} (\begin{array}{cccc} 1.00 & 0.59 & 0.80 & 0.80\\ 0.59 & 1.00 & 0.80 & 0.80\\ 0.80 & 0.80 & 1.00 & 0.77\\ 0.80 & 0.80 & 0.77 & 1.00 \end{array}). \end{array}$$ (38)

That is, the matrix of the correlations between the marginal random effects (i.e., the random effects related to the two dependent variables) is

$$\begin{array}{c} \text{Cor}\left(\gamma_{1},\gamma_{2}\right)=(\begin{array}{cc} 0.80 & 0.80\\ 0.80 & 0.80 \end{array}) \end{array}$$ (39)

whereas the estimate (on one of the previous simulated data) of this matrix, Cor(*γ*_1_, *γ*_2_), of the correlations between the marginal random effects, is

$$\begin{array}{c} \hat{\text{Cor}}\left(\gamma_{1},\gamma_{2}\right)=(\begin{array}{cc} 0.77 & 0.78\\ 0.90 & 0.74 \end{array}) \end{array}$$ (40)

If we decide to test *H*_0_: Cor(*γ*_1_, *γ*_2_) = **0** against *H*_1_: Cor(*γ*_1_, *γ*_2_) ≠ **0** in the case of these simulated data, we must know the distribution of the LR statistic *S*. In order to approximate the distribution of *S*, under *H*_0_, we proceed to an extensive simulation study in the next paragraph.

#### Empirical distribution of *S* under *H*_0_

In this paragraph, our goal is to investigate about the empirical law of the LR statistic *S*, under *H*_0_, when the size *N* of the data set increases. The simulated data sets used in this paragraph are also of bivariate longitudinal type, with *N* the total number of observations coming from *n* subjects. We choose *N* as an arithmetic sequence ranging from 50 to 2000, where the common difference is 50. We choose *n* = *N*/5 as it is sufficient to have two observations per subject for fitting the model. When *N*/*n* = 1, the random-effects parameters and the residual variance are unidentifiable [1].

The expected (standard) asymptotic distribution of *S*, under *H*_0_, is a *χ*^2^(4). This may be explained by the fact that Cov(*γ*_1_, *γ*_2_) and its transpose, Cov(*γ*_1_, *γ*_2_)^⊤^, contain four entries, respectively, and $\bar{\Gamma }$ contains Cov(*γ*_1_, *γ*_2_) and Cov(*γ*_1_, *γ*_2_)^⊤^. Therefore, the difference between the number of entries of $\bar{\Gamma }$ which need to be estimated with $L(\theta |\text{data,}\;H_{0})$ and $L(\theta |\text{data,}\;H_{1})$, respectively, is *df* = 4. Precisely, the parameters of interest are *ρ*_*η*_1_*τ*_1__, *ρ*_*η*_1_*τ*_2__, *ρ*_*η*_2_*τ*_1__ and *ρ*_*η*_2_*τ*_2__ (see Eq (14)).

**Fig 2:**
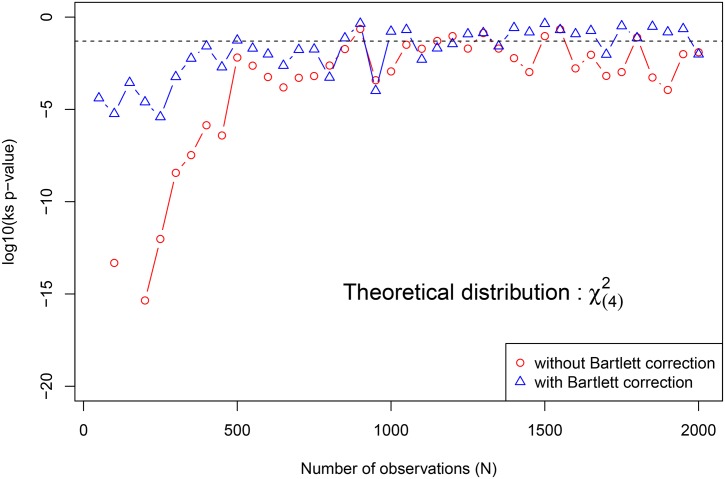
Empirical analysis of the asymptotic distribution of the LR statistic *S* under *H*_0_, using longitudinal data sets (200 replications) with size *N* ∈ {50, 100, 150, 200, …, 2000} coming from *n* ∈ {10, 20, 30, …, 400} subjects. An asymptotic distribution of *χ*^2^(4) is assumed and the Kolmogorov-Smirnov test’s p-value (at log10 scale) is ploted against the total number of observations of the data set that has served to compute the LR statistic *S*. The blue curve is obtained by applying the empirical Bartlett correction to *S* and the red curve is obtained without correction. The horizontal dashed line represents log10(0.05).

Fig 2 assumes an asymptotic distribution of *χ*^2^(4) and plots the Kolmogorov-Smirnov test’s p-value (at log10 scale) against the total number of observations of the data set that has served to compute the LR statistic *S*. The blue curve is obtained by applying the empirical Bartlett correction to *S* and the red curve is obtained without correction. The horizontal dashed line represents log10(0.05). The empirical Bartlett corrected *S*, say ${\hat{S}}_{B}$, can be expressed as ${\hat{S}}_{B}=df\times S/\hat{E}\left[S|H_{0}\right]$. This Bartlett correction is applied in order to avoid the small size distortion of the *χ*^2^(*df*) distribution, when performing the LR test using a data set of small size [78]. Fig 2 thus helps to investigate how the LR distribution performs in finite and small dimension. It also helps to investigate, in the case of this bivariate correlation test, how the Bartlett correction helps to avoid the small size distortion of the chi-square approximation. As the total number of observations increases, the curves (red and blue) reach the dashed line, gradually. Assuming the *χ*^2^(4) distribution of *S*, it seems important to work with a data set containing at least 500 observations coming from at least 2 subjects, and to apply the Bartlett correction in order to avoid the breakdown of the procedure.

**Fig 3:**
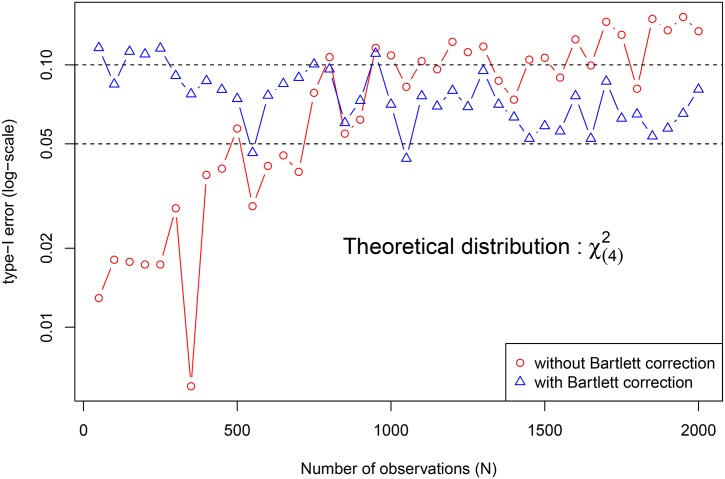
Empirical analysis of the asymptotic distribution of the LR statistic *S* under *H*_0_, using longitudinal data sets (200 replications) with size *N* ∈ {50, 100, 150, 200, …, 2000} coming from *n* ∈ {10, 20, 30, …, 400} subjects. An asymptotic distribution of *χ*^2^(4) is assumed and the type I error (at log10 scale) is ploted against the total number of observations of the data set that has served to compute the LR statistic *S*. The blue curve is obtained by applying the empirical Bartlett correction to *S* and the red curve is obtained without correction. The horizontal dashed lines represent the significance levels of 5% and 10%, respectively.

The type I error is generally controlled by the significance level of 10% (red and blue curves of Fig 3). It is clear that the control is almost full with the Bartlett correction (blue curve of Fig 3).

**Fig 4:**
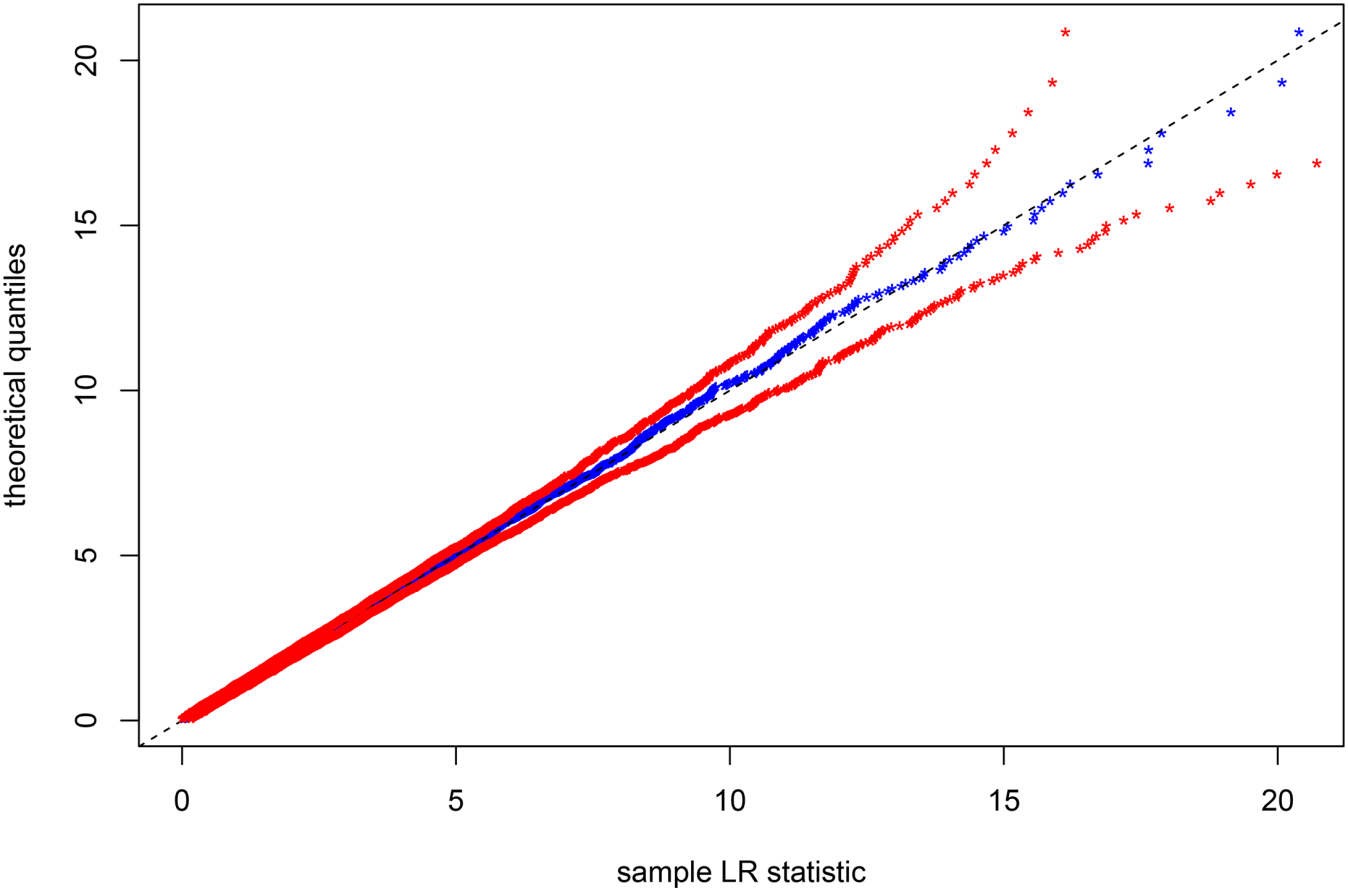
Empirical analysis of the asymptotic distribution of the LR statistic *S* under *H*_0_, using 3000 (replications) simulated longitudinal data sets (under *H*_0_) of size *N* = 15000 coming from *n* = 500 subjects. The minimum and the maximum of 1000 × 3000 simulated realizations of *χ*^2^(4) are used to construct the red sheath. The blue curve represents the LR statistics related to the bivariate correlation test.

By simulating 1000 × 3000 realizations of *χ*^2^(4) distribution, we plot the red sheath represented in Fig 4. This sheath corresponds to the minimum and the maximum of the simulated *χ*^2^(4) realizations. The blue curve inside the red sheath represents the empirical LR statistics obtained from the 3000 simulated data sets under *H*_0_. This figure (Fig 4) shows that the asymptotic distribution of LR statistic related to the bivariate correlation test is not violated, since the blue curve does not go out of the red sheath.

#### Empirical power of the bivariate correlation test

In order to analyze the power of this likelihood ratio test performed with EM-based estimates, we calculate *S* on data sets which have been simulated under *H*_0_ and *H*_1_, respectively, leading to what we named *S*0 and *S*1 vectors containing the resulting values of *S*. We then plot a ROC curve with *S*0 and *S*1, where *S*0 is the vector of the cases while S1 contains the controls. We calculate *S*0 and *S*1 in different situations where we have changed the value of *ρ* in the following configuration:

$$\begin{array}{c} Cov\left(\gamma_{1},\gamma_{2}\right)=\rho (\begin{array}{cc} \eta_{1}\tau_{1} & \eta_{1}\tau_{2}\\ \eta_{2}\tau_{1} & \eta_{2}\tau_{2} \end{array}) \end{array}$$ (41)

**Fig 5:**
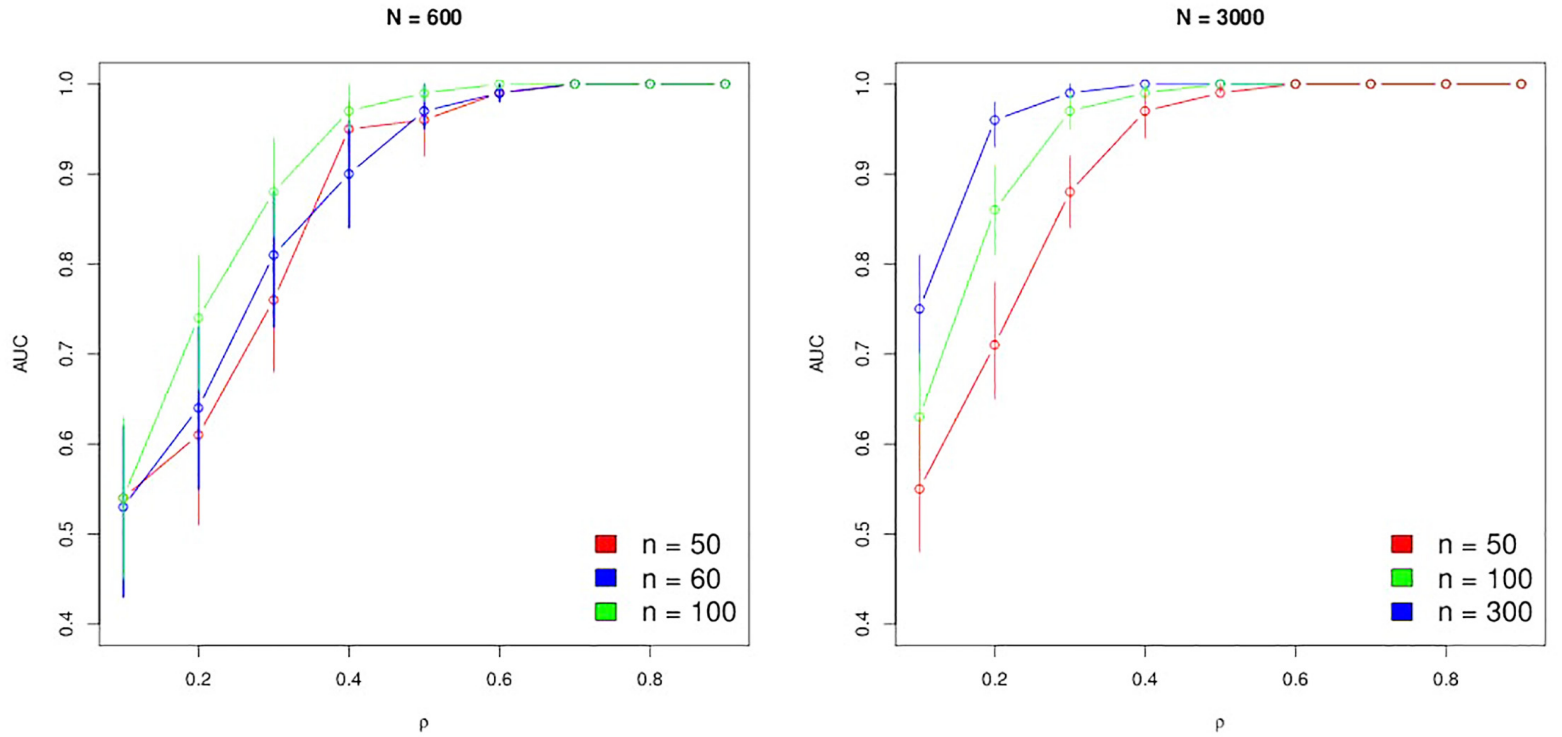
Empirical analysis of the power of the correlation test. AUC values of ROC curves with their confidence interval computed for different *ρ*, number of subjects (*n*) and observations (*N*). Left panel for *N* = 600, *n* = 50, 60, 100. Right panel for *N* = 3000, *n* = 50, 100, 300.

We maintain fixed *η*_1_ = 5.27, *η*_2_ = 6.00, *τ*_1_ = 9.89, *τ*_2_ = 1.17 and change *ρ* (∈ {0.1, 0.2, 0.3, …, 0.9}). The number of subjects (*n*) and the total number of observations (*N*) have also been modified throughout these simulation studies. In each case, the estimated Area Under Curve (AUC) of the ROC curve with its confidence interval have been recorded to produce Fig 5.

With *n* = 50 subjects, we detect, indeed, a correlation of 0.6 when the total number of observations is *N* = 3000; in contrast, if the total number of observations is *N* = 600, we perfectly detect a correlation of 0.7.

Unsurprisingly, confidence intervals of AUC are also more accurate with *N* = 3000 than with *N* = 600. With a sufficient number of observations and subjects, weak correlations are easily detected. For example, we perfectly detect a correlation of 0.2 with *N* = 3000 and *n* = 300 where AUC = 0.96(0.93 − 0.98) according to Fig 5. However, we detect quite well a correlation of 0.3 with *N* = 600 and *n* = 60 where AUC = 0.81(0.73 − 0.88).

**Fig 6:**
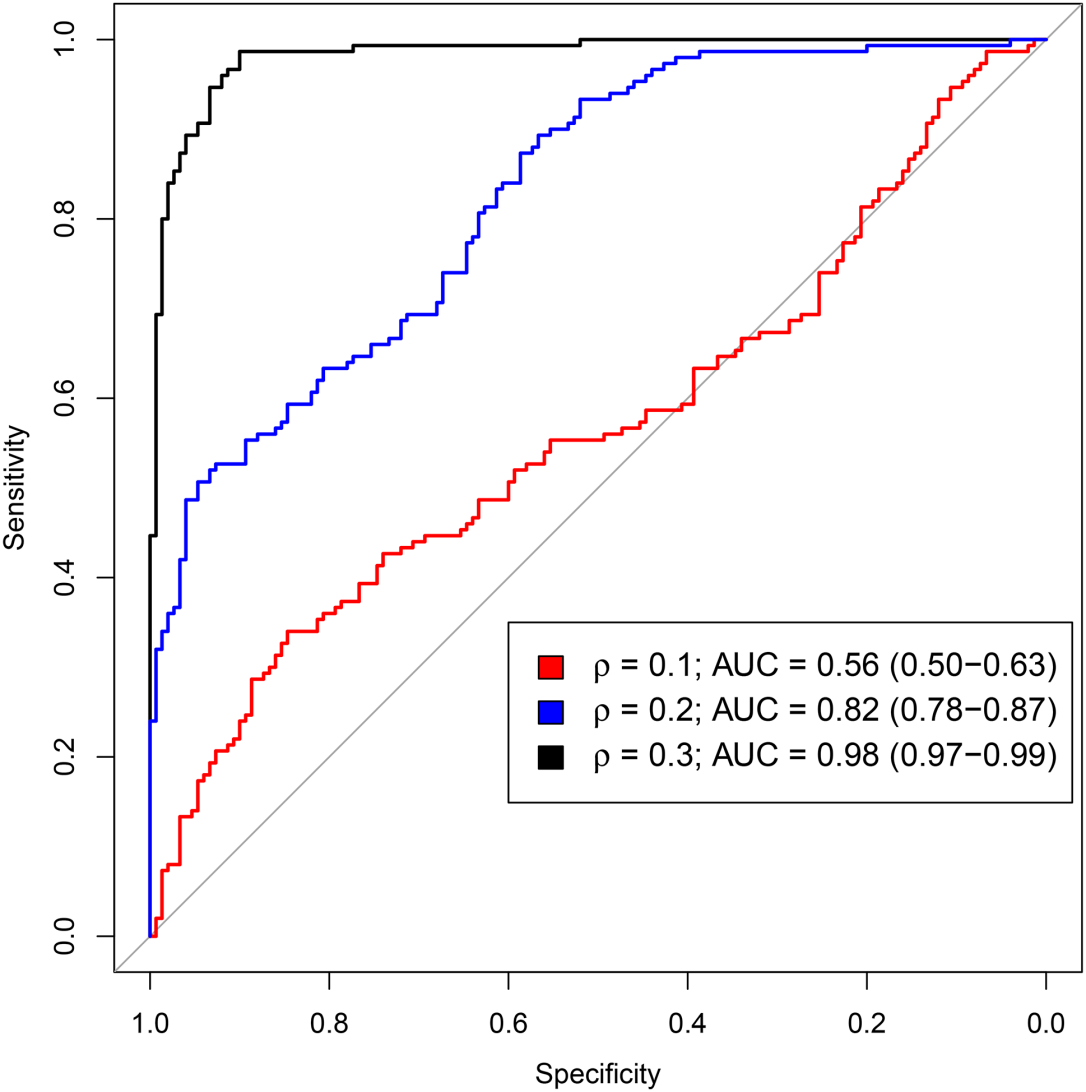
Analysis of the power of the likelihood ratio test performed under EM-estimators. ROC curves with *ρ* ∈ {0.1, 0.2, 0.3}. *N* = 5000 observations, *n* = 300 subjects, 95% CI on AUC.

In the case where estimates are of a higher quality (because they are performed on data sets having a sufficient number of observations *N* = 5000 and subjects *n* = 300), we plot ROC curves with low values of *ρ* (0.1, 0.2 and 0.3). We then show in Fig 6, the estimated AUC and its 95% confidence interval.

Fig 6 shows that EM-based estimators lead to a good power of the bivariate correlation test, when we have a sufficient number of observations and subjects in the longitudinal study case. This goodness of the power of the bivariate correlation test persists when the correlation between marginal random effects is low (about 0.2).

### Applications on real data sets

In this section we analyze two data sets by using the likelihood ratio test through the EM-based estimators presented above. The first dataset is of multivariate multilevel type and the second is, specifically, of longitudinal multivariate type.

#### Application to education data in the Netherlands

The data used here are named ‘bdf’ under the package nlme [13] of the R software. These data contain *N* = 3776 Grade eight students (aged about eleven years) in *n* = 208 elementary schools in the Netherlands [79]. These pupils were tested twice (with an interval of one year between grade seven and grade eight) on their proficiency in Dutch language and arithmetics, where the goal was to investigate which characteristics of schools can account for the differences in the effectiveness of schools with regard to pupils’ progress in language and arithmetics. Most of the previous analyses of this dataset were concerned with investigating how the language test score depends on the pupil’s intelligence, his family’s socio-economic status and on related class or school variables. By fitting two independent (separate) models, [79] found that variables in Table 4 have a significant effect on post-test scores (language post-test and arithmetic post-test). These variables are: socio-economic status, intelligence score, age, gender and nationality. They also found a significant random slope related to the language pre-test and to the gender in the language post-test model.

**Table 4:** Modeling of covariates on post-test achievement in language and arithmetic from [79].

Model	Language (post)	Arithmetic (post)
Language pre-test	0.567	-
Arithmetic pre-test	-	0.413
Socio-economic status	0.143	0.132
Intelligence score	0.124	0.270
Age	−0.069	−0.081
Gender (female)	0.187	−0.103
Ethnicity (foreign)	n.s.	−0.105

Based on these results from [79] and some of their data (*n* = 131 schools, *N* = 2287 pupils; age and ethnicity are not present), we have fitted the bivariate linear mixed-effect model where post-test scores are the response variables and covariates are the pre-test scores, socio-economic status, intelligence score, gender and minority (a factor indicating if the pupil is a member of a minority group). Random intercepts and random slopes related to pre-test scores are integrated to the model on the school level in the configuration shown by the Table 5.

**Table 5:** Model configuration.

	Variables	
Model parts	Language (post)	Arithmetic (post)
Fixed effects	Language (pre)	Arithmetic (pre)
	Socio-eco. status	Socio-eco. status
	Intelligence score	Intelligence score
	Gender	Gender
	Minority	Minority
Random effects (school level)	1	1
	Language (pre)	Arithmetic (pre)

Table 6 contains estimated fixed effects and residual standard deviations of the model.

**Table 6:** Estimated fixed effects and residual standard deviations in the joint bivariate model fitted to school data.

	Response variables			
	Language (post)		Arithmetic (post)	
Covariates	Estimate	p-value	Estimate	p-value
Intercept	4.690		−1.456	
Language (pre)	0.795	0.000	-	-
Arithmetic (pre)	-	-	0.807	0.000
Socio-eco. status	0.101	0.000	0.089	0.000
Intelligence score	0.474	0.000	0.810	0.000
Gender (female)	1.778	0.000	−0.522	0.003
Minority (yes)	−0.302	0.589	−0.545	0.194
*σ*_1_ and *σ*_2_	5.356	4.066		

The estimated covariance matrix $\hat{\Gamma }$ of the random effects is:

$$\begin{array}{c} \hat{\Gamma }=(\begin{array}{cccc} 15.15 & -0.54 & 18.20 & -0.27\\ -0.54 & 0.02 & -0.65 & 0.01\\ 18.20 & -0.65 & 30.69 & -0.48\\ -0.27 & 0.01 & -0.48 & 0.01 \end{array}) \end{array}$$

The null hypothesis, *H*_0_, that the arithmetic post-test score and the language post-test score are independent, is rejected with a p-value of 1.436 × 10^−7^. This result justifies a joint analysis of post-scores conditionally on the covariates present in the model and is therefore a supplementary information obtained from the data. The estimated correlation matrix of the random effects is:

$$\begin{array}{c} \hat{\rho }=(\begin{array}{cccc} 1.00 & -1.00 & 0.84 & -0.82\\ -1.00 & 1.00 & -0.84 & 0.82\\ 0.84 & -0.84 & 1.00 & -1.00\\ -0.82 & 0.82 & -1.00 & 1.00 \end{array}) \end{array}$$

Table 6 shows that covariates which are significant in the independent models are also significant in the joint model. The Minority covariate is neither significant in the joint model, nor significant in the independent models. ${\hat{\Gamma }}_{ij}$ and ${\hat{\rho }}_{ij}$ identify the item which is at the intersection of the row *i* and the column *j* of the matrices $\hat{\Gamma }$ and $\hat{\rho }$, respectively. These matrices are filled from the top to the bottom in the order of (Intercept_*y*_1__, Slope_*y*_1__, Intercept_*y*_2__, Slope_*y*_2__).

There is a clear inter-school variability with respect to the post-test scores (${\hat{\Gamma }}_{11}=15.15$ and ${\hat{\Gamma }}_{33}=30.69$). Everything else being equal, schools that have good scores in arithmetics also have good scores in language (${\hat{\rho }}_{31}=0.84$). The schools in which the differential effect of the pre-test score in arithmetics on the post-test score is strongly negative are in average above the average post-test score in language (${\hat{\rho }}_{41}=-0.82$); same as with the language pre-test score (${\hat{\rho }}_{32}=-0.84$). This confirms that the scores in language and in arithmetics vary in the same direction in schools. The schools in which the differential effect of the pre-test score (in arithmetics or language) on the post-test score is strongly negative are in average above the average post score (${\hat{\rho }}_{21}=-1$ and ${\hat{\rho }}_{43}=-1$), and vice versa. These schools have strived to bring the level of all pupils above the average. In contrast, pupils with a good initial level maintain their level without becoming excellent. The differential effect of the pre-test score has a very weak variability (arithmetics score: ${\hat{\Gamma }}_{22}=0.02$; language score: ${\hat{\Gamma }}_{44}=0.01$) and this implies that the pre-test score explains about 0.15% (in arithmetics) and 0.03% (in language) of inter-school variability of post-test scores.

We have fitted the bivariate model without random slopes (with random intercept only) to investigate if it fits more to the data than the model with random slopes, due to the weak variability of these random slopes. The results are presented in Table 7, where the estimated fixed effects and their significance generally remain the same.

**Table 7:** Results of the model with random intercepts only.

	Response variables			
	Language (post)		Arithmetic (post)	
Covariates	Estimate	p-value	Estimate	p-value
Intercept	4.698		−1.446	
Language (pre)	0.789	0.000	-	-
Arithmetic (pre)	-	-	0.789	0.000
Socio-eco. status	0.103	0.000	0.093	0.000
Intelligence score	0.480	0.000	0.809	0.000
Gender (female)	1.788	0.000	−0.526	0.002
Minority (yes)	−0.391	0.489	−0.498	0.249
*σ*_1_ and *σ*_2_	5.384	4.091		

The estimated covariance matrix, related to results contained in Table 7 of random effects is

$$\begin{array}{c} \hat{\Gamma }=(\begin{array}{cc} 5.15 & 5.41\\ 5.41 & 7.07 \end{array}) \end{array}$$ (42)

which indicates a correlation of $\hat{\rho }=0.895$ between the random marginal intercepts, confirming a strong positive correlation between post-test scores in arithmetics and language. With a p-value of 8.505 × 10^−5^, the likelihood ratio test indicates that the data are more compatible with the model incorporating random intercepts and random slopes at a time.

**Fig 7:**
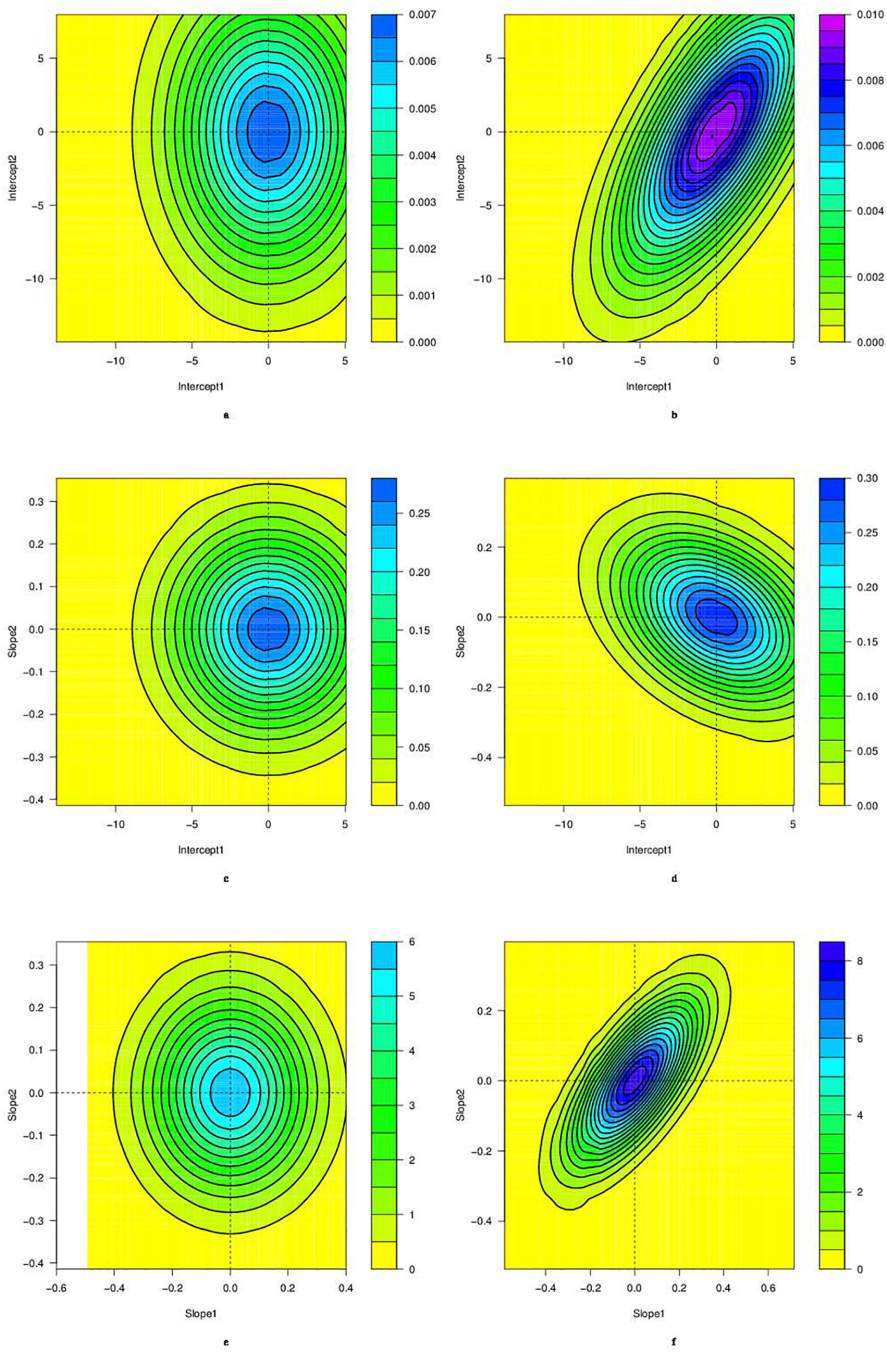
Posterior distributions of random intercepts conditional on the data related to School 47 in the education dataset. Left panels assume independence across the two dimensions while right panels assume dependence. Top panels for the joint distribution of the random intercepts, middle panels for the joint distribution of random intercept in first dimension and random slope in the second dimension, bottom panels for the joint distribution of the random slopes.

Fixed effects seem very strong and do not significantly change between the independent and bivariate models. In contrast, a posterior distribution of random effects changes significantly between the independent model and the joint bivariate model. For example, we plot the joint distribution of random effects conditional on the data concerning School 47 in the education dataset under the independent model and the joint bivariate model. Fig 7a shows the joint posterior distribution of random intercepts under the independent model whereas Fig 7b presents the same posterior distribution under the joint bivariate model. A clear difference appears between these two distributions. We notice the same difference between distributions of random intercepts and slopes as shown in Fig 7c and 7d as well as the joint distribution of random slopes in Fig 7e and 7f. The joint bivariate model seems to fit more to the present data and we retain it for their analysis.

#### Application to malaria immune response data in Benin

The data come from a study which was conducted in nine villages (Avamé centre, Gbédjougo, Houngo, Anavié, Dohinoko, Gbétaga, Tori Cada Centre, Zébè and Zoungoudo) of Tori Bossito area (Southern Benin), where *P. falciparum* is the most common species in the study area (95%) [80] from June 2007 to January 2010. The aim of this study was to evaluate the determinants of malaria incidence in the first months of life of child in Benin. Details of the follow-up procedures have been published elsewhere [81].

#### Data description

Mothers (*n* = 620) were enrolled at delivery and their newborns were actively followed-up during the first year of life. One questionnaire was conducted to gather information on women’s characteristics (age, parity, use of Intermittent Preventive Treatment during pregnancy (IPTp) and bed net possession) and on the course of their current pregnancy. Maternal peripheral blood as well as cord blood were collected into Vacutainer^®^ EDTA (Ethylene diaminetetraacetic acid) tubes. At birth, newborn’s weight and length were measured by midwives and gestational age was estimated using the Ballard method [82].

During the follow-up of newborns, axillary temperature was measured weekly. Symptomatic malaria cases, defined as fever (>37.5°C) with TBS and/or RDT positive, were treated with an artemisinin-based combination therapy as recommended by the Benin National Malaria Control Program. Systematically, TBS were made every month to detect asymptomatic infections. Every three months, venous blood was sampled to quantify the level of antibody against malaria promised candidate vaccine antigens. The environmental risk of exposure to malaria was modeled for each child, derived from a statistical predictive model based on climatic, entomological parameters, and characteristics of children’s immediate surroundings as reported by [83].

Concerning the antibody quantification, two recombinant *P. falciparum* antigens were used to perform IgG subclass (IgG1 and IgG3) antibody quantification by Enzyme-Linked ImmunoSorbent Assay (ELISA) standard methods developed for evaluating malaria vaccines by the African Malaria Network Trust (AMANET [www.amanet148trust.org]). Protocol was described in detail [84].

#### Data analysis

For our analysis, we use some of the data and we rename the proteins used in the study described above, for confidentiality reasons (some important findings are yet to be published). Thus, the proteins we use here, are named A1, A2, B and C, and are related to the antigens IgG1 and IgG3 as mentioned above in the description of the study. A1 and A2 are different domains of the same protein A, and C and D are two different proteins. Information contained in the multivariate longitudinal dataset of malaria are described in the Table 8, where Y denotes a protein which is one of the following:

$$\begin{array}{c} \text{IgG1}\_\text{A1},\text{IgG3}\_\text{A1},\text{IgG1}\_\text{A2},\text{IgG3}\_\text{A2},\text{IgG1}\_\text{B},\text{IgG3}\_\text{B},\text{IgG1}\_\text{C},\text{IgG3}\_\text{C} \end{array}$$ (43)

**Table 8:** Variables present in the analyzed dataset.

Variable	Description
id	Child ID
conc.Y	concentration of Y
conc_CO.Y	Measured concentration of Y in the umbilical cord blood
conc_M3.Y	Predicted concentration of Y in the child’s peripheral blood at 3 months
ap	Placental apposition
hb	Hemoglobin level
inf_trim	Number of malaria infections in the previous 3 months
pred_trim	Quarterly average number of mosquitoes child is exposed to
nutri_trim	Quarterly average nutrition scores

The aim of the analysis of these data is to evaluate the effect of the malaria infection on the child’s immune (against malaria). Since the antigens which characterize the child’s immune status interact together in the human body, we analyze the characteristics of the joint distribution of these antigens, conditional on the malaria infection and other factors of interest. The dependent variables are then provided by conc.Y (Table 8) which describes the level of the protein Y in the children at 3, 6, 9, 12, 15 and 18 months. All other variables in the Table 8 are covariates. We then have eight dependent variables which describe the longitudinal profile (in the child) of the proteins listed in Eq (43).

In the models that we fit to these data, we specify one random intercept by child and one random slope by child in the direction of the malaria infection. The illustration we do here is to jointly analyze each of the 28 pairs of proteins, in order to investigate if some profiles of proteins are independent, conditional on the configuration of the fitted model. After performing the bivariate correlation test on all 28 bivariate models, the obtained p-values, with a Bonferroni correction, range from 4.16 × 10^−33^ to 0.932. The p-value 0.932 is the only one which is not significant. This p-value corresponds to the pair of proteins (IgG3_A1, IgG1_B).

**Fig 8:**
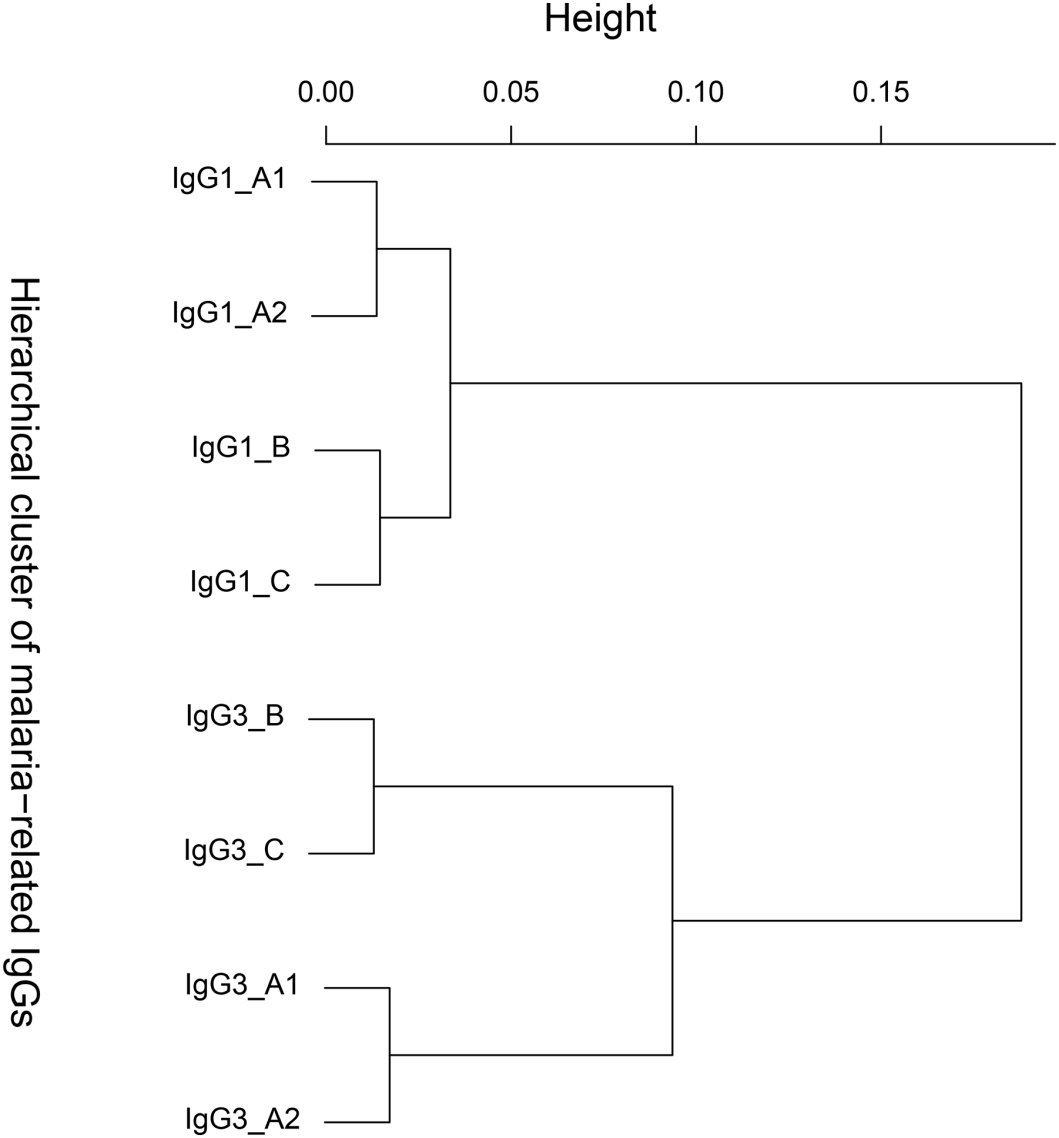
Hierarchical cluster tree on malaria-related proteins.

To investigate the general configuration of these proteins, in terms of correlations, we build their hierarchical cluster tree using −log(p-value) as dissimilarity. This hierarchical cluster tree is presented by the Fig 8.

The branch related to the IgG1 is different from the branch related to the IgG3. In other words, IgG1_A1, IgG1_A2, IgG1_B and IgG1_C are on the same branch which is different from the branch containing IgG3_A1, IgG3_A2, IgG3_B and IgG3_C (Fig 8). Relatively to both IgG1 and IgG3, A1 and A2 go together, and B and C also go together. These results are biologically very consistent, since A1 and A2 are domains of the same protein, and B and C are two different proteins. On the cluster (Fig 8), it also appears that the proteins IgG3_A1 and IgG1_B which are not significantly correlated (according to our bivariate test) are distant. Statistically, the model which may be used to jointly analyze these 8 protein profiles is not probably the model which contains all the 27 significant correlations, avoiding overfitting problems. Based on the results provided by the bivariate correlation test, it may be useful to perform a regularization procedure in the fitting of the full eight-variate model.

## Conclusion

In the context of the multivariate linear mixed-effects model, we have suggested the more general expressions of the EM-based estimators than those used in the literature to analyze multivariate longitudinal data. These estimators fit the framework of the multivariate multilevel data analysis which, obviously, englobes the multivariate longitudinal data analysis framework. We also have built a likelihood ratio test based on these EM estimators to test the independence of two dimensions of the model. Furthermore, the simulation studies have validated the power of this test and have shown that this is an extremely sensitive test. In the context of longitudinal data, it allows to detect a modest correlation signal with a very small sample (*ρ* = 0.3, AUC = 0.81, with *n* = 60 subjects and *N* = 600 observations). In the simulation studies, the empirical distribution of the likelihood ratio statistic fits the *χ*^2^(4). The asymptotic properties of likelihood ratio statistics, under nonstandard conditions, have been shown by [85] and [86]. These works have been generalized by [87] to cover a large class of estimation problems which allow sampling from non identically distributed random variables. The asymptotic distribution of the LR statistic derived by [87] is a mixture of chi-squared distributions. In the context of likelihood ratio tests for variance components in linear mixed-effects models, [88] used the results of [87] to prove that the proposed mixture of chi-squared distributions is the actual asymptotic distribution of such LR used as test statistics for null variance components with one or two random effects. Based on these works, Further theoretical investigations may be done to properly find out the asymptotic distribution of the likelihood ratio statistic in the case of this bivariate correlation test. Finally, we have illustrated the usefulness of the test on two different real-life data. The first dataset, which is of multivariate multilevel type, concerns the effects of school and classroom characteristics on pupils’ progress in Dutch language and arithmetics, where the scores in language and arithmetics are the two response variables which have been considered. Our method has yielded results that are consistent both with information in existing publications and with a conceptual understanding of the phenomenon. On this dataset, we have highlighted a joint effect between the scores in arithmetics and language within schools in the Netherlands. The second dataset, which is of longitudinal multivariate type, concerns a study of the effect of the malaria infection on the child’s immune response in Benin. By jointly analyzing all the pairs of protein profiles of interest, we have plotted a hierarchical cluster tree of these proteins, using the bivariate correlation test. Information contained in this hierarchical cluster tree is consistent with the biological literature related to this issue.

The model as it is written is easily extendable to more dimensions despite a sparsity problem in choosing the parameterization of the covariance matrix or the precision matrix. Probably we could use this two-dimensional dependence test to structure a larger covariance matrix. The bivariate correlation test can help to construct iteratively, using a stepwise procedure, a parsimonious joint model containing all the components of ***y***. This stepwise procedure may consist in adding to the constructing model, at each step, the significant correlation between two dependent variables. Using a model selection strategy, the model which fits more to the data will be retained. It could possibly be advantageous to turn to graphical LASSO type approaches to make a penalized estimation of this covariance (or precision) matrix. We could also resort to the rapid optimization methods such as that implemented in the lme4 [76] package, given the slow pace of the EM algorithm. It would be useful to assess the interest of this method compared to some heuristics such as the one which consists in setting one marginal response variable as a covariate of the other(s).

## Supporting Information

S1 File Empirical data sets used in the applications section. (RDATA)

S2 File R script used to perform the simulation studies. (R)

S3 File Functions used in the R script. (R)

S4 File Estimated likelihood ratio statistics under H0 hypothesis. These statistics help to plot the blue curve in Fig 4.
(RDATA)

S5 File Statistics of the bivariate correlation test performed on the multivariate longitudinal data related to malaria. These statistics help to construct the hierarchical tree of the malaria protein profiles.
(RDATA)
